# Bacteria Associated With *Shiraia* Fruiting Bodies Influence Fungal Production of Hypocrellin A

**DOI:** 10.3389/fmicb.2019.02023

**Published:** 2019-09-11

**Authors:** Yan Jun Ma, Li Ping Zheng, Jian Wen Wang

**Affiliations:** ^1^College of Pharmaceutical Sciences, Soochow University, Suzhou, China; ^2^Department of Horticultural Sciences, Soochow University, Suzhou, China

**Keywords:** *Shiraia* fruiting body, associated bacteria, diversity, hypocrellin A, co-culture

## Abstract

Hypocrellin A (HA) is a natural red perylenequinone pigment from *Shiraia* fruiting body, which was used clinically on various skin diseases and developed as a photodynamic therapy agent against cancers. The fruiting bodies may harbor a diverse but poorly understood microbial community. In this study, we characterized the bacterial community of *Shiraia* fruiting body using a combination of culture-based method and Illumina high-throughput sequencing, and tested the involvement of some companion bacteria in fungal HA production using the fungal–bacterial confrontation assay. Our results revealed that the bacterial community in the fruiting body was dominated by *Bacillus* and *Pseudomonas*. Some *Pseudomonas* isolates such as *P. fulva*, *P. putida*, and *P. parafulva* could stimulate fungal HA accumulation by *Shiraia* sp. S9. The bacterial treatment of *P. fulva* SB1 up-regulated the expression of polyketide synthase (*PKS*) for HA biosynthesis and transporter genes including ATP-binding cassette (*ABC*) and major facilitator superfamily transporter (*MFS*) for HA exudation. After the addition of live *P. fulva* SB1, the mycelium cultures of *Shiraia* sp. S9 presented a higher HA production (225.34 mg/L), about 3.25-fold over the mono-culture. On the other hand, *B. cereus* was capable of alleviating fungal self-toxicity from HA *via* down-regulation of HA biosynthetic genes or possible biodegradation on HA. To our knowledge, this is the first report on the diversified species of bacteria associated with *Shiraia* fruiting bodies and the regulation roles of the companion bacteria on fungal HA biosynthesis. Furthermore, the bacterial co-culture provided a good strategy for the enhanced HA production by *Shiraia*.

## Introduction

*Shiraia bambusicola* P. Hennings is a pathogenic fungus of bamboos, and its fruiting body has been used in traditional Chinese medicine for the treatment of vitiligo, stomachache, psoriasis, and rheumatic pain (Zhen and Di, 1995). The red perylenequinone pigments isolated from the fruiting body including hypocrellin A (HA), B, and C have attracted intense interest as new non-porphyrin photosensitizers in photodynamic therapy (PDT) for cancers and human immunodeficiency virus (Di et al., 1990; Mulrooney et al., 2012). Due to the difficulty in their chemical synthesis (O’Brien et al., 2010), the fruiting bodies are the main resources for hypocrellin supply in medical application (Liang et al., 2009).

In the wild, the fungal fruiting bodies grow and develop in association with various endophytic bacteria. The predominant aerobic bacteria *Pseudomonas fluorescens* were found in fruiting body of *Cantharellus cibarius* (Danell et al., 1993). The cultivable bacteria such as *Sphingomonas paucimobilis* and *P. aeruginosa* were associated with the fruiting bodies of different ectomycorrhizal fungi (Dahm et al., 2005). Varese et al. (1996) characterized the companion bacteria from fruiting body and ectomycorrhizae of *Suillus grevillei*, and found that *Streptomyces* could inhibit the growth of its mycobiont but *Pseudomonas* could enhance the fungal growth. Sbrana et al. (2000) reported that *Pseudomonas* strains isolated from *Tuber borchii* ascocarp were able to produce some antimycotic metabolites. Both *Bacillaceae* and *P. fluorescens* from the stroma of *T. borchii* could degrade cellulose and chitin for possible modification of fruiting body (Citterio et al., 2001). However, the presence and role of the bacteria associated with *Shiraia* fruiting bodies remain unknown. As the bacteria from fungal fruiting bodies presented a possible regulation on fungal growth and metabolite production, we herewith wish to explore the bacterial community of *Shiraia* fruiting body. The bacteria were screened and evaluated for their ability to regulate HA production in the *Shiraia* mycelium cultures. The co-cultures between live bacterium and its fungal host *Shiraia* were established for HA production. To our knowledge, this is the first report on the diversity of bacteria associated with *Shiraia* fruiting bodies and their roles in HA biosynthesis.

## Materials and Methods

### Sampling Collection and Observation of the Fruiting Bodies

The fruiting bodies were picked from the bamboo (*Brachystachyum densiflorum*) twigs at their intermediate developing stage from June to July 2016 in Tianmu mountain of Hangzhou, China, and collected from three plots (approximately 100 m^2^/plot). Each sample contained 30 fruiting bodies. All samples were immediately stored in sterilized sealed bags on ice. In order to observe the structural features of the fruiting body, the samples were cut into thin slices and observed with a light microscope (CKX41, Tokyo, Japan). The paraffin wax sections of fruiting bodies were made according to the method reported by Lyon et al. (1995). The morphology of the fruiting body was further observed using a scanning electron microscope (SEM, S-4700, Hitachi, Japan).

### Isolation and Screening of HA-Producing Fungus

Fresh fruiting bodies were sterilized by immersion in 0.1% HgCl_2_ (w/v) for 3 min and washed with sterile water. Fungi derived from the fruiting body were obtained according to the method described by Chen et al. (2004). Isolated pure fungi were preserved on potato dextrose agar (PDA) slant culture. To screen HA-producing fungal strain, the isolated fungus was inoculated into a 150-ml Erlenmeyer flask containing 50-ml potato dextrose broth (PDB) and cultured in a rotary shaker at 150 rpm at 28°C for 8 days (Yang et al., 2013). The mycelia were then harvested by centrifugation at 8,000 rpm for 10 min and dried at 60°C overnight. The dried mycelia were powdered and extracted with acetone for 10 h. The rapid identification of perylenequinonoid derivatives in the acetone extracts was performed using a chemical color response test (Wan and Chen, 1981). To further confirm the presence of HA in mycelium, we measured the UV-VIS absorption spectrum using a Shimadzu UV-2600 (Kyoto, Japan) spectrophotometer and mass spectrum using Agilent 6120 HPLC–MS system (Wilmington, DE, United States). Finally, strain S9 was selected on the basis of higher hypocrellin yield (Supplementary Table S1) and identified as *Shiraia* sp. through the observation of its morphology as well as the ITS rDNA sequence (GenBank Accession No. MF062656.1) (see section “Results”). The strain has been deposited in China General Microbiological Culture Collection Center (CGMCC) with the deposition number CGMCC16369 and was cultured routinely and stored at 4°C on PDA medium.

### HA Quantification

The extraction and determination of HA were based on the methods described in our previous report (Sun et al., 2017). HA content was determined by the reverse-phase Agilent 1260 HPLC system (Agilent, Co., Wilmington, DE, United States) equipped with the Agilent HC-C18 column (250 mm × 4.6 mm dimension) with a mobile phase (acetonitrile: water at 65: 35, v/v) for 20 min at 1 ml/min at 465 nm. The HA was quantified with the standard (Chinese National Compound Library, Shanghai, China). Total HA production refers to the sum of the intracellular and extracellular HA. Total HA production was calculated according to the following equation: Total HA production (mg/L) = intracellular HA content (mg/g DW) × fungal biomass (g DW/L) + extracellular HA production (mg/L). Values are mean ± SD from three independent experiments.

### Isolation and Identification of Bacteria Associated With Fruiting Bodies

The bacteria were isolated from 15 fruiting bodies (5 fruiting bodies per replicate) by using surface sterilization method (Kumari et al., 2013). The internal tissue of the sporocarps was ground and the dilutions of the tissue extracts were plated on Luria-Bertani (LB) agar and glucose-peptone-yeast (GPY) medium containing cycloheximide (100 μg/ml) at 30°C for 1–4 days (Zagryadskaya et al., 2013). The bacterial isolates were successively transferred and restreaked until pure cultures were obtained.

Total DNA of bacteria were extracted from the overnight bacteria culture, and DNA integrity and purity were confirmed using the Agilent 2100 Bioanalyzer (Agilent Technologies, Santa Clara, CA, United States). The 16S rDNA was amplified using the conserved bacterium-specific primers, 27F (5′-AGAGTTTGATCATGGCTCAG-3′)/1492R (5′-TACG GCTACCTTGTTACGACTT-3′) (Li et al., 2016a). The PCR reaction was performed in a final volume of 25 μl, comprising 200 ng of hyphal DNA, 0.5 μl of 10 × dNTPs, 0.5 μl of 27F (5 nmol/L), 0.5 μl of 1492R (5 nmol/L), and 0.125 μl of Taq DNA polymerase, made up to the final volume with double distilled water (dd H_2_O). The reaction was performed under the following conditions: 3 min at 94°C, followed by 30 cycles of 30 s at 94°C, 30 s at 56°C, and 1 min at 72°C by using T-100 thermal cycler (Bio-Rad, Hercules, CA, United States). Sequencing of the amplified product was carried out by Sangon Biotech, Co., Ltd. (Shanghai, China). The phylogenetic tree of bacteria was constructed by using phylogeny server^1^ and the neighbor-joining method.

### Biochemical Characteristics of the Bacteria

The essential biochemical characteristics including Gram character, glucose utilization, starch hydrolysis, citrate utilization, nitrate reduction, oxidase, catalase, and gelatin liquefaction of bacteria have been analyzed and tested (Kreig and Holt, 1984).

### Library Preparation and 16S rDNA Bacteria Deep Sequencing

Total genomic DNA of fruiting body was extracted as described above. Two sets of primers to amplify the 16S rDNA gene of bacteria were applied (Wu et al., 2015). The universal primers 338F (5′-ACTCCTACGGGAGGCAGCA-3′) and 806R (5′-GGACTACHVGGGTWTCTAAT-3′) were used to amplify the V3–V4 hypervariable regions of 16S rDNA genes. The universal primers 515F (5′-GTGCCAGCMGCCGCGG-3′) and 907R (5′-CCGTCAATTCMTTTRAGTTT-3′) were used to amplify the V4–V5 hypervariable regions of 16S rDNA genes. The PCR reaction were performed in a 50-μl volume containing 5 μl of 10 × Ex Taq buffer, 1 μl of 10 mM dNTP Mix, 2 μl of 10 mM each forward and reverse primers, 1 μl (200 ng) of template DNA, and 38.7 μl of dd H_2_O. All reactions were performed under the following conditions: 3 min at 95°C, followed by 32 cycles of 30 s at 95°C, 30 s at 56°C, and 15 s at 72°C. A final 5-min extension step at 72°C was also performed. The PCR products were confirmed using 2% (w/v) agarose gel. The DNA band with the correct size was excised and purified using a PCR Clean-up System (Omega Bio-Tek, Doraville, GA, United States). After purification, the PCR products of 16S rDNA V3–V5 regions were quantified by Pico Green analysis (Molecular Probes, Eugene, OR, United States). The libraries were then sequenced using Illumina MiSeq platform (Illumina, San Diego, CA, United States) (Caporaso et al., 2012).

### Processing and Analyzing of the Sequencing Data

Raw sequence data (NCBI Accession No. PRJNA541247) were sorted based on sample-specific barcode tags and then the primers and tag sequences were trimmed from sorted sequences. The resultant sequences were processed initially using the Trimmomatic (Anthony et al., 2014) and FLASH programs (Reyon et al., 2012) to obtain paired-end (PE) reads. The qualified sequences were clustered into operational taxonomic units (OTUs) defined at 97% identity using CD-HIT program (Li and Godzik, 2006). The taxonomic assignment of sequence was classified using Greengenes database (DeSantis et al., 2006) and RDP classifier at 50% threshold (Wang et al., 2007). The analyses of rarefaction curve and alpha diversity index were performed using Mothur program (Schloss et al., 2009).

### Fungal–Bacterial Confrontation Assay

An *in vitro* confrontation bioassay between the fungus S9 and bacteria was conducted using the reported method by Wang et al. (2015). The stock culture of S9 strain was maintained on PDA slant at 4°C and the strain was initially grown on PDA medium in a Petri dish at 28°C for 8 days. A small piece (5 mm × 5 mm) of the strain from the PDA plate was dug and placed in the center of a 10-cm PDA plate for 4 days. To analyze the effect of live bacterium on mycelia growth and hypocrellin secretion of S9 strain preliminarily, the single colony of bacterium was inoculated in LB broth (without agar) at 37°C on a rotary shaker at 200 rpm for 12 h. Then, bacterial suspension (10 μl) was streaked in two parallel straight lines, approximately 7 cm apart from each other. After incubation for 10 days, the S9 colony morphology was observed and photographed. The S9 strain without any treatment was used as blank control.

### The Establishment of Co-cultures

The co-culture was established between the live bacterium and *Shiraia* sp. S9 in the mycelium cultures. A small piece (5 mm × 5 mm) of the strain from PDA plate was dug and transferred into a 150-ml Erlenmeyer flask containing 50-ml PDB broth at 28°C on a rotary shaker at 150 rpm for 4 days. The single colony (approximately 2 mm diameter) of bacterium was inoculated in LB broth (without agar) at 37°C on a rotary shaker at 200 rpm for 12 h. Then, bacterial cells were added into the cultivation medium of *Shiraia* at a final density of 100 cells/ml. After 8 days of co-cultures at 28°C on a rotary shaker at 150 rpm, the mycelia were harvested and dried to constant weight in a 60°C oven to evaluate the fungal biomass and HA contents.

### The Determination of HA Toxicity, Bacterial Susceptibility, and Degradation

HA (purity > 98%, Chinese National Compound Library, Shanghai, China) was dissolved in absolute ethanol at 1 g/L as stock solution. The minimum inhibitory concentration (MIC) of HA on fungal growth was determined by broth dilution susceptibility testing method (Singh et al., 2010). HA at 0.0–2.2 mg/L was applied into the fungal spore suspension (800 μl of 1 × 10^5^ spores/ml) in a 1.5-ml reaction tube, which was cultured in the dark on a rotary shaker at 150 rpm and 28°C for 8 days. To further analyze the bacterial effect on the MIC value of HA, bacterial cells (400 μl of 0.75 × 10^3^ cells/ml) were added to the test system. To test the bacterial degradation of HA, the bacterial cells (400 μl of 0.75 × 10^3^ cells/ml) in LB mixed with 60 mg/L HA in a 5-ml reaction tube were incubated in the dark on a rotary shaker at 150 rpm and 28°C according to the description of Mitchell et al. (2002). HA quantification was carried out using the method described above. Finally, the bacterial susceptibility to HA was also examined. Bacterial cells (400 μl of 0.75 × 10^3^ cells/ml) were incubated in LB with HA at 0–100 mg/L in a 5-ml reaction tube on a rotary shaker at 150 rpm and 28°C for 24 h in the dark. The bacterial cell density was measured using a UV-VIS spectrophotometer (UV-2600, Shimadzu, Japan) at 600 nm.

### Quantitative Real-Time PCR Analysis

After 24-h co-culture with live bacteria (SB1 and No. 1), total RNA of the fungal mycelia was extracted using RNAprep pure Plant Kit (Tiangen, Beijing, China). The primers of target genes (Supplementary Table S2) for HA biosynthesis and internal reference gene (18S ribosomal RNA) were designed with the Primer Express software (Applied Biosystems, Foster City, CA, United States). The qRT-PCR condition and procedure were set and performed as our previous report (Sun et al., 2017).

### Statistical Analysis

All treatments consisted of triplicate independent repeats (10 plates or flasks per replicate). Student’s *t*-test and one-way analysis of variance (ANOVA) with Dunnett’s multiple comparison tests were applied for experimental results. All results were expressed as mean ± standard deviation (SD), with *p* < 0.05 being considered statistically significant.

## Results

### The Morphology and Structure of the Fruiting Body

**FIGURE 1:**
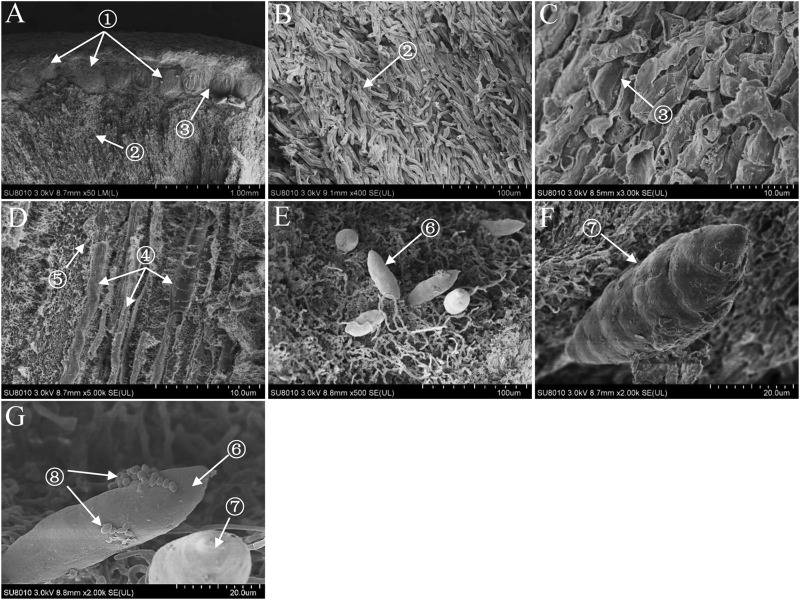
Scanning electron micrographes **(A–G)** of transverse section of *Shiraia* stroma. ① Perithecium; ② pseudoparenchyma; ③ prosenchyma; ④ ascus; ⑤ paraphyses; ⑥ immature ascospore; ⑦ mature ascospore; ⑧ bacterium.

The fruiting body of *S. bambusicola* presented irregular pinkish tubercles surrounding branches of the host bamboo (Supplementary Figures S1A–C). The perithecia with a flask shape were located in the edge of the fruiting body (Supplementary Figure S1D). The germination and development of mature ascospores with vertical-tranverse cystidium were found in both ascocarp (Supplementary Figure S1E) and the substrate of the fruiting body (Supplementary Figure S1F). In addition, a significant number of conidia were produced near the stalk of bamboos during the tubercle expansion and moved onto the stromal surface. As shown in Supplementary Figures S1G,H, we found clearly that the asci arranged in the perithecia in parallel. Within the ascus, immature ascospores were produced and spread. Meanwhile, the abundant paraphyses were found around the asci (Supplementary Figure S1I). The observation based on SEM revealed that the fruiting body of *S. bambusicola* was mainly composed of pseudoparenchyma and prosenchyma, and the latter was only distributed around the ascocarp (Figures 1A–C). It was obvious that the asci were distributed parallel to each other in ascocarp (Figure 1D). The generation of ascospores was found in the substrate of stroma (Figure 1E). There were several clear transverse cystidia on the surface of mature ascospore (Figure 1F). We observed that a certain number of bacteria were on the surface of mature ascospore (Figure 1G).

### Isolation and Identification of HA-Producing Fungus

**FIGURE 2:**
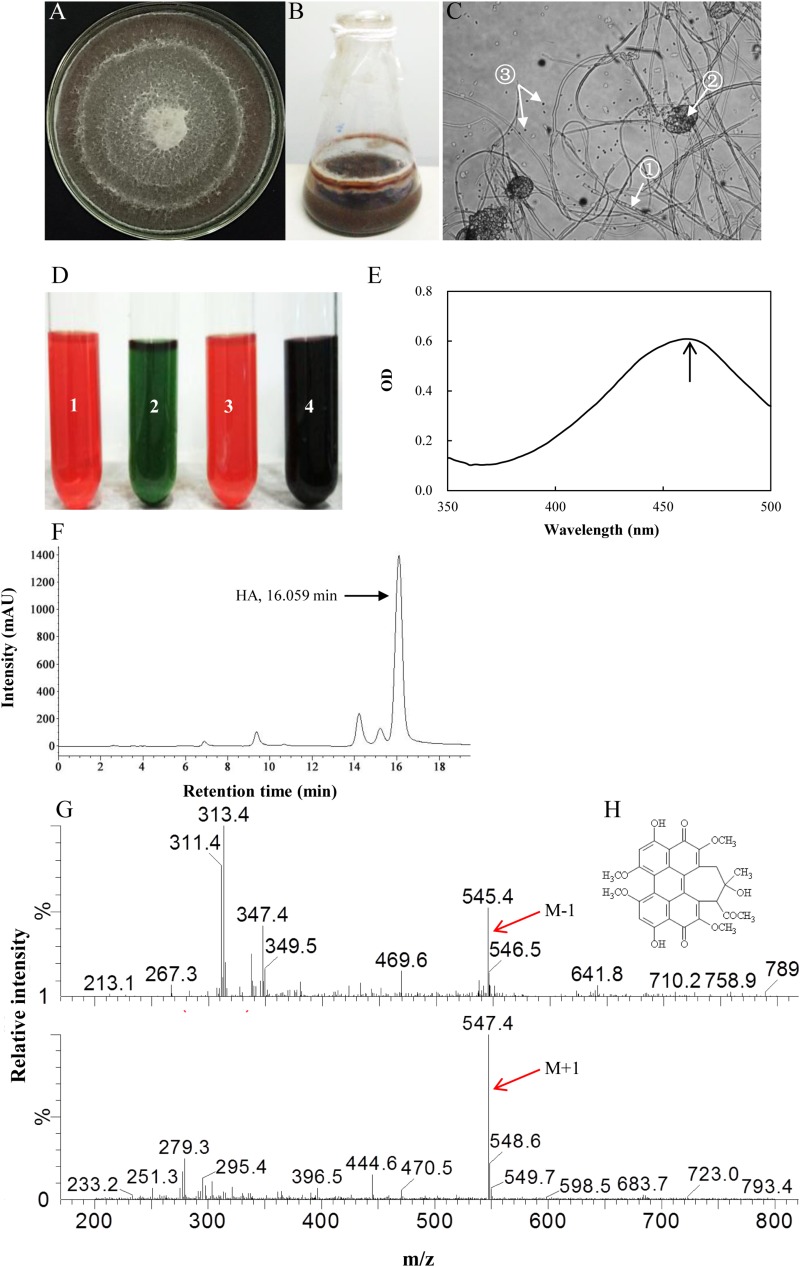
Characterization of hypocrellin production by strain S9. **(A)** The observation of the red pigment secretion on day 10 of the strain on PDA. **(B)** The fermentation of the strain in PDB for 4 days. **(C)** The morphological structure of the strain under optical microscope (100×). ① Septal hypha; ② pycnidium; ③ conidium. **(D)** The typical color reaction. Pigment acetone extract (1) with 1 mol/L sodium hydroxide solution (2). Pigment acetone extract with 1 mol/L hydrochloric acid solution (3). Pigment acetone extract with 1 mol/L FeCl_3_ solution (4). **(E)** Full wavelength scanning of the strain acetone extracts. **(F)** The retention time of HA of wild *Shiraia* sp. S9 by HPLC. **(G)** Qualitative analysis of HA by LC–MS. M-1, *m/z* [M-H] 545.4, negative ion spectra with one adduct proton. M + 1, *m/z* [M + H] 547.4, positive ion spectra with one adduct proton. **(H)** The structure of HA. Molecular formula: C_30_H_26_O_10_.

Six red pigment-producing fungi were isolated from the fruiting body, and the strain S9 was chosen on the basis of higher hypocrellin yield on PDA plate (Supplementary Table S1). The mycelia of S9 could accumulate red pigment in the both PDA plate (Figure 2A) and liquid flask culture (Figure 2B). The S9 strain has septal hyphae and several pycnidia, which could generate a large number of conidia (Figure 2C). To validate the hypocrellin production by S9 strain, the rapid identification of perylenequinone was carried out by using a series of typical color reactions (Wan and Chen, 1981). As shown in Figure 2D, the liquid of acetone extract with sodium hydroxide (1 in Figure 2D) was red and then turned green under alkaline condition (2 in Figure 2D). The pigment was red under acid condition (3 in Figure 2D) and became dark purple when FeCl_3_ was added into the liquid (4 in Figure 2D). Simultaneously, the pigment exhibited a typical characteristic absorption peak of perylenequinones at 465 nm (Figure 2E) (Wan and Chen, 1981). To further confirm the structure of HA, LC–MS analysis was performed after the compound was purified (Figure 2F). Figure 2G shows the ESI-MS spectrum with the [M-H] at *m/z* 545.4 and the [M+H] at *m/z* 547.4, indicating that the molecular weight is 546.4. According to the chromatographic characteristics and previous report (Liang et al., 2009), we conferred that the compound was HA with the molecular formula C_30_H_26_O_10_ (Figure 2H). Subsequently, a length of 522 bp sequence (Supplementary Figure S2A) was amplified from the genome DNA of S9 strain with the ITS1/ITS4 as primers, and the sequence has been submitted to NCBI^2^. The S9 strain (GenBank Accession No. MF062656.1) was initially classified as *Shiraia* sp. based on hit taxon strain with the ITS rDNA sequence data in the Basic Local Alignment Search Tool (BLAST) server^3^ compared to *Shiraia* sp. SUPER-H168 (GenBank AccessionNo. EU267793.1) with a similarity of 94%. A phylogenetic tree of S9 based on ITS rDNA sequences is shown in Supplementary Figure S2B using the phylogeny server. Together, we concluded that the fungus S9 was proved to belong to the genus *Shiraia*.

### The Bacteria Associated With the Fruiting Bodies

**FIGURE 3:**
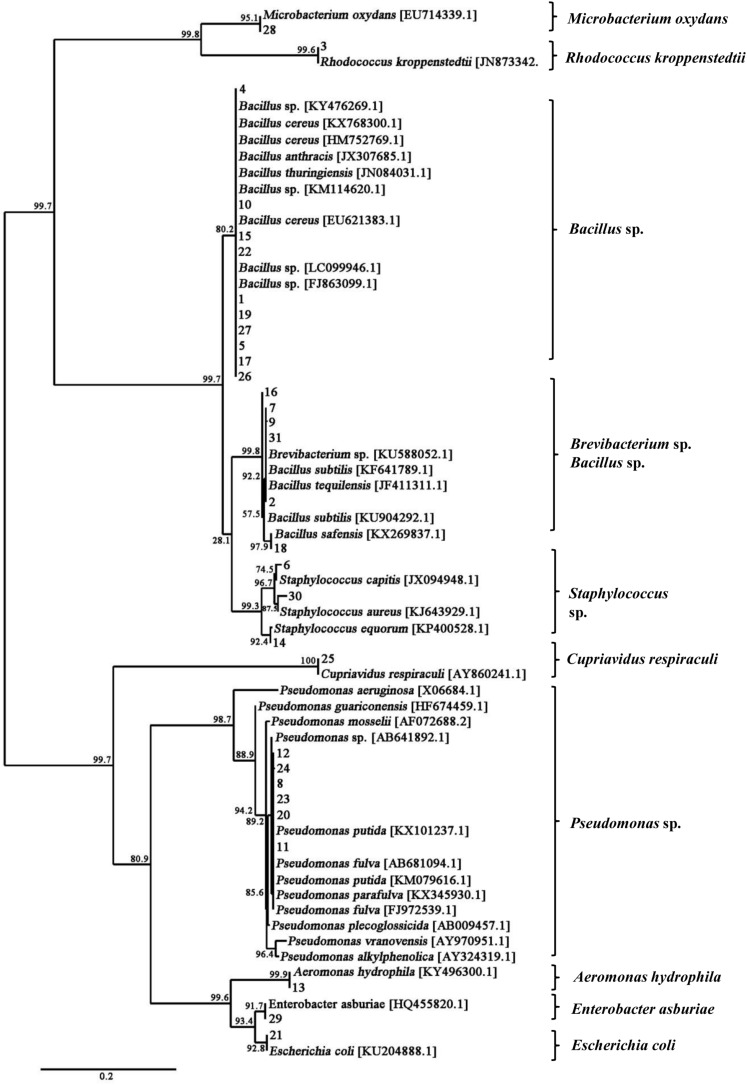
Phylogenetic tree of 16S rDNA gene sequences showing the relationships between the bacteria isolated from the fruiting body and the reference strains. The branch length is proportional to the number of substitutions per site. Bar: 0.2 substitutions per nucleotide position.

A total of 31 bacterial strains were isolated from the fresh fruiting body of *S. bambusicola* (Supplementary Figure S3). Sequencing and comparison of the PCR products of 16S rDNA revealed that the bacterial isolates belonged to 3 phyla, 4 classes, 6 orders, 9 families, 10 genera, and 17 species (Supplementary Table S3). Moreover, the phylum of Firmicutes (approximately 58%) and its class of Bacilli (55%) were the predominant taxa among the isolates. Based on full-length 16S rDNA sequences, the phylogenetic analysis differentiated total bacteria into different clusters at the genus level (Figure 3). Supplementary Table S4 shows that the dominating bacterial genera were *Bacillus* (approximately 45%) and *Pseudomonas* (approximately 20%). *Bacillus* spp. (14 strains) showed the average similarities to *B. cereus* (96–98%), *B. tequilensis* (97%), *B. subtilis* (97%), *B. safensis* (98%), and *B. anthracis* (97%). In addition to the *Bacillus* isolates, six bacterial strains were identified as *Pseudomonas* spp., displaying high similarities to the species of *P. putida* (96–97%), *P. fulva* (97–99%), and *P. parafulva* (96%). Three bacterial isolates (Nos. 6, 14, and 30 in Supplementary Table S4) were assigned as *Staphylococcus* spp. with 97, 96, and 96% similarities to the *S*. *capitis*, *S*. *equorum*, and *S*. *aureus*, respectively. Two isolates (Nos. 2 and 9 in Supplementary Table S4) represented *Brevibacterium* spp. with a similarity of 97%, and the remaining six strains represented other bacterial species. The biochemical characteristics of these bacteria are presented in Supplementary Table S5. Gram-positive rods were the predominant type of bacteria isolated from the fruiting bodies, with the exception of *Pseudomonas* spp., *Escherichia coli*, *Cupriavidus respiraculi*, and *Enterobacter asburiae*.

### The Bacterial Communities in the Fruiting Bodies

**FIGURE 4:**
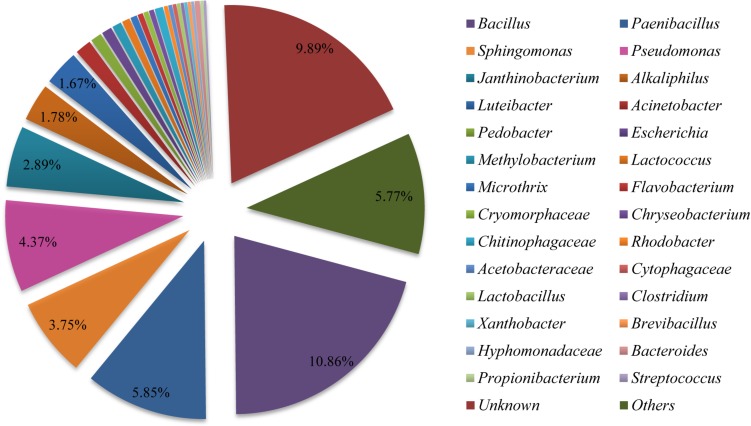
Relative distribution of the bacterial genera in *Shiraia* fruiting body. Sequences are binned at the genus level and the abundance values are depicted as the percentage of the total bacterial sequences in a sample.

In order to determine the more abundant composition of the bacterial communities in the fruiting body, purified PCR products were subjected to Illumina Miseq library and sequenced. A total of 37,610 clean reads were obtained from the sample examined in this study (Supplementary Table S6). We found 723 OTUs (Supplementary Tables S6, S7) in the bacterial community of the sample based on 97% species identity. The Shannon Wiener index and Chao index values were 4.468 and 806.817, respectively (Supplementary Table S6). The sequence clustered into 723 bacterial OTUs that belonged to 30 bacterial phyla, 84 classes, 149 orders, 244 families, and 364 genera (including unclassified) (Supplementary Tables S7–S9). The phylum of Proteobacteria (approximately 38%) and Firmicutes (approximately 13%) were the predominant taxa among the isolates (Supplementary Table S9). As shown in Figure 4, the relative distribution of bacteria revealed that most of OTUs showed similarity to *Bacillus* (approximately 10.86%), *Paenibacillus* (5.85%), *Pseudomonas* (4.37%), *Sphingomonas* (3.75%), *Janthinobacterium* (2.89%), *Alkaliphilus* (1.78%), and *Luteibacter* (1.67%) at the genus level, which were partly consistent with those found in fruiting body by using culture-dependent approach (Supplementary Table S4).

### Effects of Live Bacterium on Fungal HA Production

After the fungus–bacteria confrontation assay, we found that some *Pseudomonas* isolates such as *P. putida* (No. 8), *P. fulva* (No. 11), and *P. parafulva* (No. 20) exhibited the capacity to stimulate the secretion of red pigments from the mycelium (Supplementary Figure S4). Among them, No. 11 named *P. fulva* SB1 showed the most significant effect on red pigment accumulation. In the presence of live SB1, HA content in PDA plate reached 6.18 mg/cm^2^, which is 2.09-fold higher than that of the control group (Supplementary Table S10). The SB1 strain colonies appeared round, smooth, flat to convex in shape, and creamy yellow in color (Supplementary Figure S5A). A phylogenetic tree of *P. fulva* SB1 based on 16S rDNA sequences (GenBank Accession No. MF058662.1) is shown in Supplementary Figure S5B using the phylogeny server. Simultaneously, some bacterial isolates such as *Aeromonas hydrophila* (No. 13) and *Microbacterium oxydans* (No. 28) have no obvious effects on pigment secretion, while other *Bacillus* isolates suppressed the accumulation of pigment remarkably (Supplementary Figure S4 and Supplementary Table S10). There was no detection of HA production under the influence of five *Bacillus* strains (Nos. 1, 15, 18, 19, and 22 in Supplementary Table S10).

**FIGURE 5:**
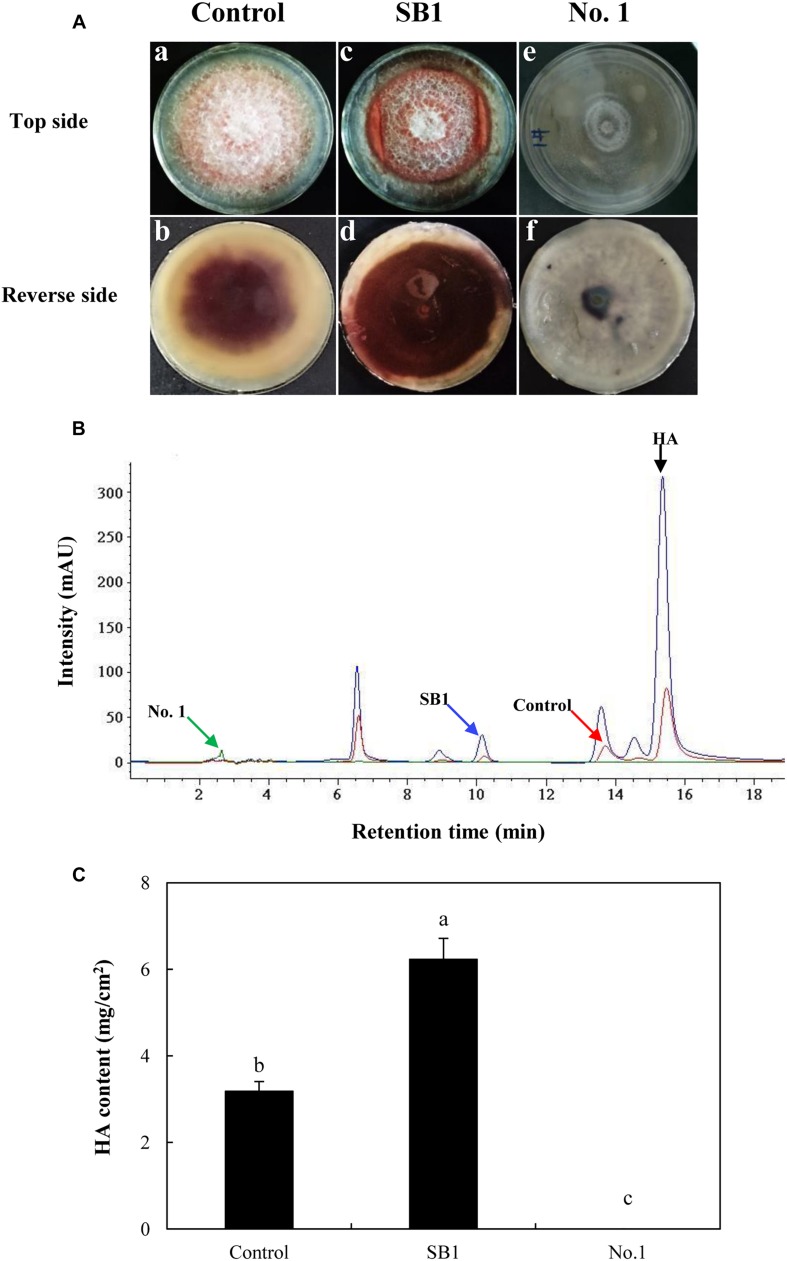
**(A)** The effect of live bacteria (*P. fulva* SB1 and *B. cereus* No. 1) on the growth and red pigment secretion of *Shiraia* sp. S9. The blank control group without treatment (a and b). The treatment group treated with live SB1 cells (c and d) and No. 1 cells (e and f). **(B)** The retention time of HA of *Shiraia* sp. S9. **(C)** The HA content of *Shiraia* sp. S9 in PDA plate. A small piece (5 mm × 5 mm) of the strain was placed in the center of a 10-cm PDA plate at 28°C for 4 days. The single colony of bacterium was inoculated in LB at 37°C on a rotary shaker at 200 rpm for 12 h. Then, bacterial suspension (10 μl) was streaked in two parallel straight lines on PDA at 28°C for 10 days, approximately 7 cm apart from each other. Values are mean ± SD from three independent experiments. Different letters above the bars mean significant differences (*p* < 0.05).

As shown in Figure 5A, after the incubation without live bacterial cells for 10 days, the S9 strain presented abundant aerial mycelia and certain red substrate mycelia (Control in Figure 5A). Compared to the control group, the live SB1 stimulated the secretion of red pigment into medium significantly lacking white aerial mycelia (SB1 in Figure 5A), while the hyphal growth and pigment accumulation was significantly suppressed by *B. cereus* No. 1 (No. 1 in Figure 5A, GenBank Accession No. MH734616). Subsequently, the influence of live bacterial cells on HA production in solid PDA plate was determined using HPLC (Figure 5B). Incubation under SB1 cells resulted in an increase in HA content by 95.91%, compared with the control group, while *B. cereus* No. 1 cells curbed the HA biosynthesis completely (Figure 5C).

**FIGURE 6:**
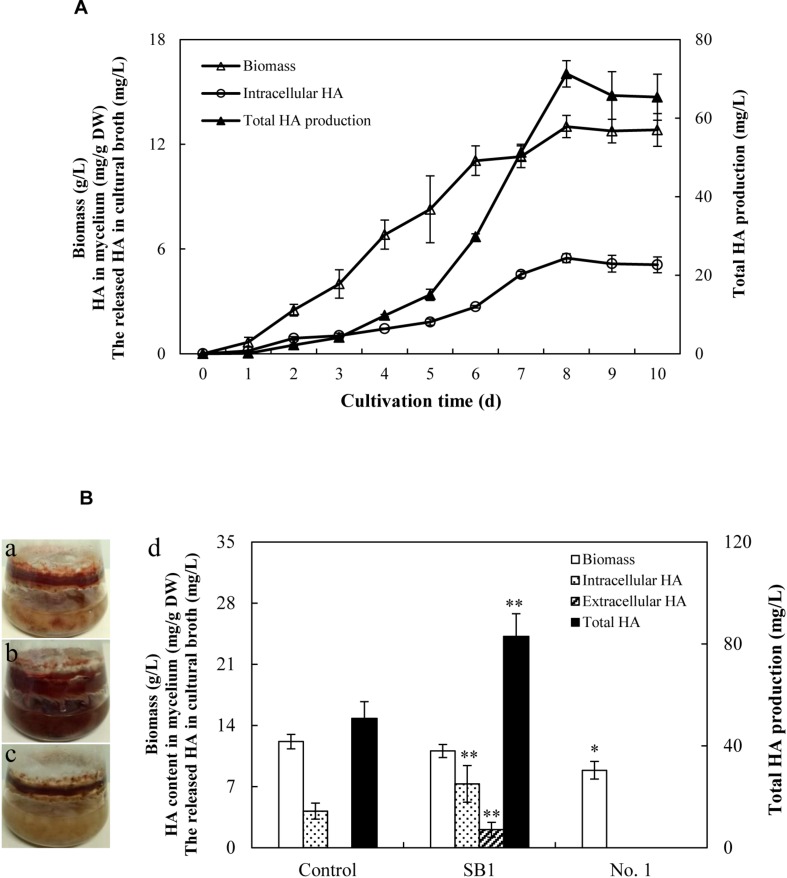
**(A)** Time profiles of growth and HA accumulation in submerged culture of S9 strain. **(B)** Observation of submerged cultures of *Shiraia* sp. S9 with or without live bacterial cells on day 8. **(a)** Control group. **(b)** Live *P. fulva* SB1 treatment. **(c)** *B. cereus* No. 1 treatment. **(d)** The effect of live bacteria on hyphal biomass, intracellular HA, extracellular HA, and total HA production in submerged cultures of *Shiraia* sp. S9. The bacteria at 100 cells/ml were added to the cultures on day 4. Values are mean ± SD from three independent experiments. ^∗^*p* < 0.05, ^∗∗^*p* < 0.01 versus control group.

Due to the limitation of the wild sources of the fruiting bodies of *S. bambusicola*, the submerged mycelium culture is becoming a promising alternative for HA production (Yang et al., 2009). In the submerged mycelium cultures of S9, the strain has a typical time course of hypha growth and HA production (Figure 6A). The hypha biomass showed an exponential growth between 1 and 5 days. Moreover, the hypha biomass reached the maximum weight of 13.01 g/L on day 8. The production of total HA increased to the highest value of 71.27 mg/L with time up to day 8 accordingly, and then decreased appreciably. However, no release of HA was detected in PDB broth during the submerged culture. Subsequently, the influence of live bacterial cells on HA production was investigated in liquid cultures (Figure 6B). The live bacteria of *P. fulva* SB1 and *B. cereus* No. 1 at a density of 100 cells/ml was applied to the submerged culture of *Shiraia* sp. S9 on day 4 at 150 rpm and 28°C for 8 days, separately. As shown in Figure 6B, compared with the control (Figure 6B-a, without bacterial cells addition), the secretion of red pigment was increased by live SB1 treatment (Figure 6B-b) but suppressed by *B. cereus* No. 1 cells obviously (Figure 6B-c). In submerged cultures, the changes on HA content and total production were similar to that in solid culture plates. The live SB1 promoted HA production to 82.98 mg/L on day 8, a 1.63-fold of the control group (Figure 6B-d). It was noteworthy that *P. fulva* SB1 stimulated not only intracellular HA biosynthesis in mycelium, but also the released HA (2.05 mg/L) in medium (Figure 6B-d).

### Effect of Live Bacterium on Expression of HA Biosynthetic Genes

**FIGURE 7:**
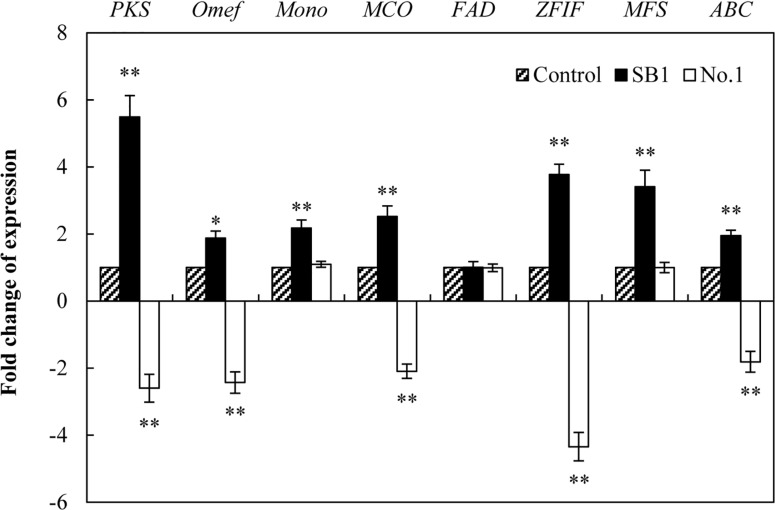
The effect of live bacteria on the expression of *Shiraia* HA biosynthetic genes. The bacteria SB1 and No. 1 at 100 cells/ml were added to the cultures on day 4 for 24 h, respectively. *PKS*, polyketide synthase; *Omef*, *O*-methyltransferase; *Mono*, monooxygenase; *MCO*, multicopper oxidase; *FAD*, FAD/FMN-dependent oxidoreductase; *ZFIF*, zinc finger transcription factor; *MFS*, major facilitator superfamily; *ABC*, ATP-binding cassette transporter. Values are mean ± SD from three independent experiments. ^∗^*p* < 0.05, ^∗∗^*p* < 0.01 versus control group.

To investigate the regulation mechanism of live bacterium on HA biosynthesis, some HA biosynthesis-related genes (Zhao et al., 2016) including polyketide synthase (*PKS*), *O*-methyltransferase (*Omef*), monooxygenase (*Mono*), multicopper oxidase (*MCO*), FAD/FMN-dependent oxidoreductase (*FAD*), zinc finger transcription factor (*ZFIF*), major facilitator superfamily (*MFS*), and ATP-binding cassette transporter (*ABC*) were analyzed by using qRT-PCR after 24 h of the co-culture. The expressions of all tested genes except *FAD* gene were up-regulated by SB1 in the co-culture, about 5.49-, 1.88-, 2.18-, 2.53-, 3.78-, 3.41-, and 1.95-fold of the mono-culture control, respectively (Figure 7). On the contrary, *B. cereus* down-regulated most of these gene expressions significantly, including *PKS* (2.60-fold), *Omef* (2.43-fold), *MCO* (2.09-fold), *ZFIF* (4.34-fold), and *ABC* (1.81-fold).

### Optimization of the Co-cultures for Enhanced HA Production

**FIGURE 8:**
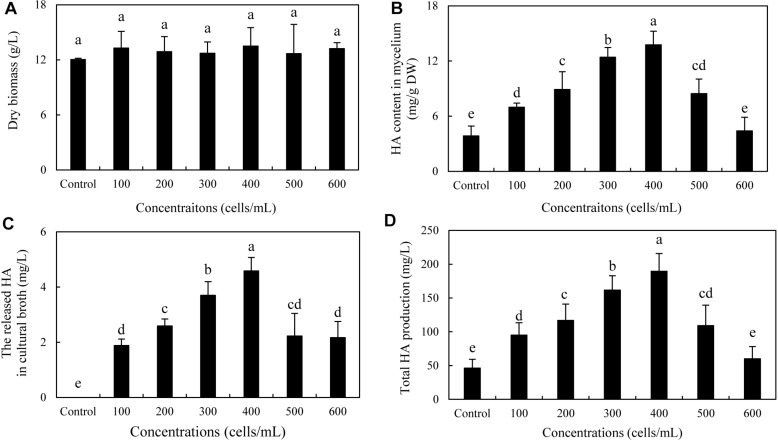
Effect of live SB1 treatment at different concentrations on mycelium dry biomass **(A)**, HA content in mycelium **(B)**, the released HA in cultural broth **(C)**, and total HA production **(D)** in submerged culture of *Shiraia* sp. S9. The culture was maintained in a 150-ml flask containing 50 ml of the liquid medium at 150 rpm and 28°C for 8 days and treated by live SB1 on day 4. The control represents the condition with no live SB1 treatment. Values are mean ± SD from three independent experiments. Different letters above the bars mean significant differences (*p* < 0.05).

**FIGURE 9:**
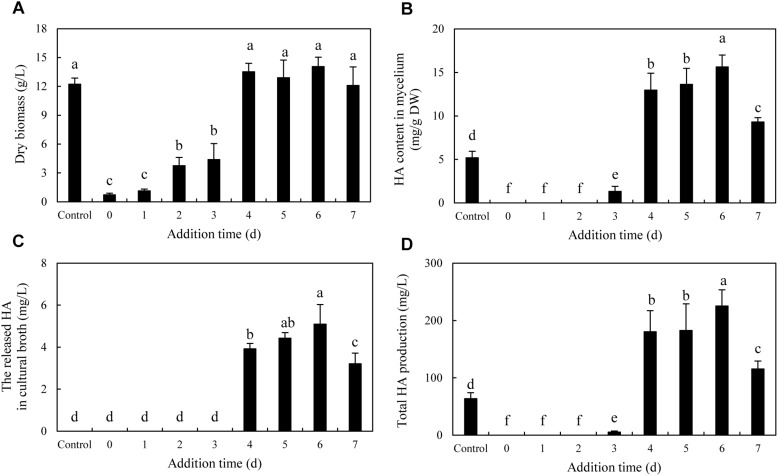
Effect of the addition time of live SB1 on mycelium dry biomass **(A)**, HA content in mycelium **(B)**, the released HA in cultural broth **(C)**, and total HA production **(D)** in submerged culture of *Shiraia* sp. S9. The *Shiraia* culture was maintained in a 150-ml flask containing 50 ml of the liquid medium at 150 rpm and 28°C for 8 days. Live SB1 was added in the *Shiraia* cultures on day 0–7 at 400 cells/ml. The control represents the condition with no live SB1 treatment. Values are mean ± SD from three independent experiments. Different letters above the bars mean significant differences (*p* < 0.05).

**FIGURE 10:**
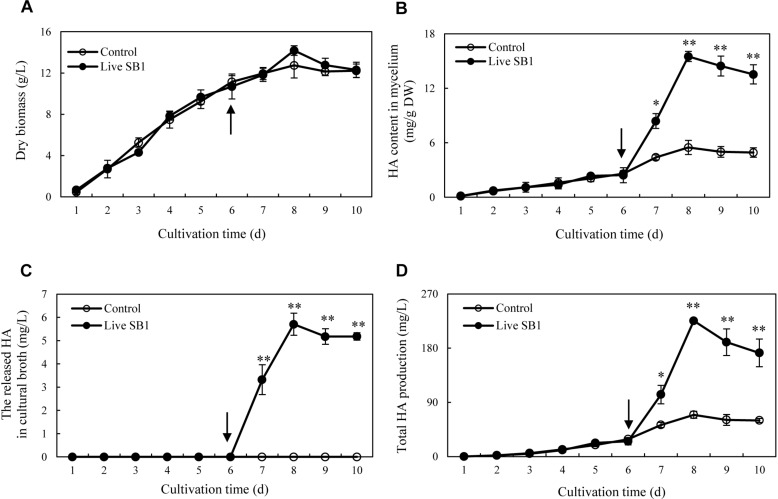
Time profiles of mycelium dry biomass **(A)**, HA content in mycelium **(B)**, the released HA in cultural broth **(C)**, and total HA production **(D)** in submerged culture of *Shiraia* sp. S9 with or without live SB1 treatment at 400 cells/ml on day 6. The culture was maintained in a 150-ml flask containing 50 ml of the liquid medium at 150 rpm and 28°C for 8 days. The *arrow* represents the time of addition of live SB1. Values are mean ± SD from three independent experiments. ^∗^*p* < 0.05 and ^∗∗^*p* < 0.01 versus control.

To optimize the co-cultures for HA production, different concentrations (0–600 cells/ml) of bacterial *P. fulva* SB1 were applied to the cultures of fungus S9. The lower concentration at a density of 100–400 cells/ml showed no retardation on fungal growth but promotion on HA biosynthesis (Figures 8A–C). The maximum HA content in mycelium reached 13.76 mg/g DW at 400 cells/ml, which is 3.57-fold of the control (Figure 8B). The release of HA in cultural medium was activated and reached its peak (4.59 mg/L) at 400 cells/ml (Figure 8C) and then decreased appreciably. The trend of total HA production (Figure 8D) was similar to HA content. To investigate the influence of addition time of SB1, the fungal biomass and HA accumulation were measured on day 8 after SB1 treatment at different addition times (0–7 days) (Figure 9). When SB1 at a density of 400 cells/ml was added on day 6 of initial fermentation, it not only promoted the accumulation of HA in hyphae (15.64 mg/g DW) but also induced the secretion of HA into the broth at 5 × 10 mg/L with the maximum total HA production on day 8. Hence, we chose the concentration of 400 cells/ml and addition time (day 6) as the optimized conditions for the co-cultures. After live SB1 treatment at a density of 400 cells/ml, mycelial biomass was not altered obviously (Figure 10A). The highest value of HA content in mycelium, released HA, and total HA production was achieved on day 8, respectively (Figures 10B–D). Total HA production in cultures was 225.34 mg/L, about 3.25-fold that of control group.

### The Toxicity and Biodegradation of HA

**FIGURE 11:**
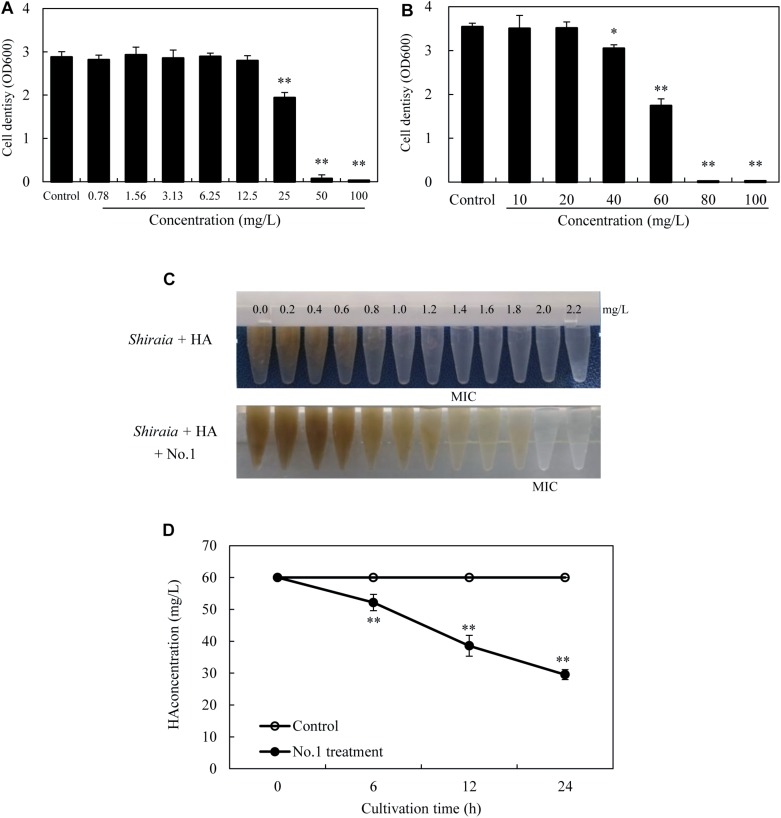
The bacterial susceptibilities to HA. *P. fulva* SB1 **(A)** or *B. cereus* No. 1 **(B)** was incubated in LB with HA at 0–100 mg/L on a rotary shaker at 150 rpm and 28°C for 24 h in the dark. **(C)** The toxicity of HA on the fungal growth of *Shiraia* sp. S9. HA at different concentrations (0.0–2.2 mg/L) was added to the fungal spore suspension (800 μl of 1 × 10^5^ spores/ml) with or without addition of No. 1 cells for 8 days. **(D)** Time profiles of HA degradation in LB with No. 1 cells. The bacterial cells (0.75 × 10^3^ cells/ml, 400 μl) mixed with 60 mg/L HA were incubated in the dark on a rotary shaker at 150 rpm and 28°C. MIC, minimum inhibitory concentration. Values are mean ± SD from three independent experiments. ^∗^*p* < 0.05, ^∗∗^*p* < 0.01 versus control group.

After incubation with HA for 24 h in the dark, no observable antibacterial activity against both bacteria was found in the presence of HA at a concentration lower than 12.5 mg/L (Figures 11A,B). The bacterial density was inhibited only at a higher concentration of HA (≥25 mg/L for SB1 and ≥40 mg/L for No. 1). However, the self-toxicity of HA on *Shiraia* growth was observed (Figure 11C). The MIC value of HA on fungal growth was 1.4 mg/L, whereas the addition of *B. cereus* No. 1 could decrease HA-induced suppression and increase MIC value to 2.0 mg/L. Furthermore, HA degrading ability of *B. cereus* No. 1 was tested (Figure 11D). After 24 h of the incubation, HA was reduced by 50.78% in the presence of strain No. 1.

## Discussion

It has been reported recently that fruiting bodies could harbor a broad spectrum of microorganisms including bacteria, yeasts, and filamentous fungi (Yurkov et al., 2012; Kumari et al., 2013). In *Shiraia* fruiting bodies, the only reported isolates were *Shiraia* spp. until now. Although *S. bambusicola* has been reported as a species of the monotypic genus *Shiraia* (Qi et al., 2015), some HA-producing *Shiraia* strains were isolated recently from the fruiting bodies or the tissues of bamboo, including *S. bambusicola* ZH-5-1 (Hu et al., 2008), *Shiraia* sp. SUPER-H168 (Liang et al., 2009), *S. bambusicola* UV-62 (Yang et al., 2009), *S. bambusicola* GZUIFR-11K1 (Du et al., 2013), and *Shiraia* sp. zzz816 (Shen et al., 2014). In the above reports, the HA production of the ZH-5-1 strain, SUPER-H168 strain, UV-62 strain, and GZUIFR-11K1 strain could reach 5 mg/g DW, 2.02 mg/g DW, 196.94 mg/L, and 12.99 mg/L, separately. In our present study, we isolated six strains of HA-producing ability from the fruiting bodies (Supplementary Table S1). The phylogenetic result further demonstrated that a high-yielding strain S9 was initially classified as *Shiraia* sp. based on ITS rDNA sequence data in the BLAST with a similarity of 94% to *Shiraia* sp. SUPER-H168 (Supplementary Figure S2). The total production of HA in mycelium cultures of *Shiraia* sp. S9 reached 71.27 mg/L on day 8 (Figure 6), suggesting that S9 is a potential fungal strain for biotechnological production of HA.

Except for the isolated fungus, we hereby found that bacteria colonized the internal part (gleba) of *Shiraia* fruiting bodies (Figure 1). Different bacteria (31 strains) were isolated from the fresh fruiting bodies (Supplementary Figure S3 and Supplementary Table S4). Among these bacteria associated with the fruiting bodies, *Bacillus* species were the most predominant bacteria (45%), followed by *Pseudomonas* (20%) and *Staphylococcus* species (10%). On the other hand, Illumina sequences provided more rapid and discriminatory analysis on the bacterial communities including 723 bacterial OTUs that belonged to 30 bacterial phyla, 84 classes, 149 orders, 244 families, and 364 genera (Supplementary Tables S6–S8). Our results demonstrated that *Shiraia* fruiting bodies have high abundance taxa of Proteobacteria and Firmicutes (Supplementary Tables S3, S9). All bacterial genera and higher-level taxa observed in culture were also detected by deep sequencing. We detected *Bacillus* and *Pseudomonas* with higher frequency based on both methods (culture-based and Illumina high-throughput sequencing), whereas members of *Paenibacillus* and *Sphingomonas* were common in high-throughput sequencing data but rare in cultures, indicating a method-driven discrepancy (Pent et al., 2017) or the possible existence of non-culturable bacteria (Kataoka et al., 2012). The dominant bacteria detected here in the fruiting bodies suggested a similarity to the ectomycorrhizosphere, where *Bacillus*, *Pseudomonas*, *Paenibacillus*, and *Sphingomonas* were common members of ectomycorrhizal fungi (Poole et al., 2001; Dahm et al., 2005). The most common bacterial communities in fruiting bodies of *Tricholoma matsutake* and *Agaricus bisporus* were also reported to be the high abundance of *Pseudomonas* and *Bacillus*, respectively (Li et al., 2016b; Xiang et al., 2017). Although some of the observed bacterial taxa including *Staphylococcus*, *Bacillus*, *Enterobacter*, and *Pseudomonas* in *Shiraia* fruiting bodies were present in bamboo endophytic bacteria, *Arthrobacter*, *Curtobacterium*, and *Alcaligenes* were found only from bamboo tissues (leaves, stems, and roots) (Yuan et al., 2015). Our results suggested the possibility that the endophytic bacteria may colonize around hyphae and make themselves as potent fruiting body associates. However, the relative abundance of these bacteria differed significantly between these two habitats. Differences between endophytic and fungal-associated bacterial communities may be related to nutritional status (Nazir et al., 2010) and the interaction with the host cells (Danell et al., 1993). On the other hand, hypocrellin extracts from the fruiting bodies possessed strong inhibition on Gram-positive bacteria such as *S. aureus*, *B. subtilis*, and *Listeria monocytogenes* under light irradiation, but no activity against Gram-negative bacteria such as *E. coli* and *Salmonella typhimurium* in a dark place or under light irradiation (Su et al., 2009). The structure of bacterial communities in the fruiting bodies may also be affected by the presence of antibacterial hypocrellins synthesized by the host fungus. Previous studies presented a prevalence of Gram-negative bacterial communities associated with the fruiting bodies of ectomycorrhizal fungi (Dahm et al., 2005; Zagryadskaya et al., 2013). However, we found that about two-thirds of isolates from the fruiting bodies were Gram-positive bacteria belonging to the genera *Bacillus*, *Brevibacterium*, *Rhodococcus*, *Staphylococcus*, and *Microbacterium* (Supplementary Table S5). Our results from bacterial susceptibility also showed that lower concentration of HA (<12.5 mg/L) had no antibacterial activity against both *B. cereus* No. 1 and *P. fulva* SB1 (Figures 11A,B). The possible resistance of those fruiting body-associated bacteria to hypocrellins needs to be investigated in the future.

To our knowledge, this is the first report to present the bacterial community in *Shiraia* fruiting body. Despite many bacterial isolates from various fruiting bodies (Li and Godzik, 2006; Zagryadskaya et al., 2013; Benucci and Bonito, 2016), the physiological roles of the bacterial associates have not been well-elucidated so far. Two *Pseudomonas* strains (DJ35 and DY22) from fruiting body of *A. bisporus* were reported to be potent mushroom growth-promoting bacteria due to their production of indole acetic acid (IAA) and cellulase (Xiang et al., 2017). The development of the primordia and basidiome of *Pleurotus ostreatus* was enhanced by some fluorescent *Pseudomonas* spp. screened from the mycelial surface (Cho et al., 2003). Sbrana et al. (2002) screened ectomycorrhizas of *T. borchii* and found that 17 isolates were able to increase mycelial growth. These studies strongly suggest that specific bacteria intimately associated with fungi have potential promotion on mycelial growth and the development of fruiting bodies. However, whether the bacteria inside the fruiting bodies have the potential to affect the biosynthesis of secondary metabolites in host fungi is still rarely known. The bacteria associated with truffle-fruiting bodies were reported to be capable of bioconversion of non-volatile precursor (such as methionine) into thiophene derivative, the principal ingredient of truffle aroma (Splivallo et al., 2015). In the present study, it is an interesting finding that the some *Pseudomonas* strains such as *P. fulva*, *P. parafulva*, and *P. putida* exhibited the capacity to stimulate the secretion of red pigments in substrate mycelia of their host *Shiraia* sp. S9 (Supplementary Figure S4 and Supplementary Table S10). The most significant enhancement in fungal HA production was obtained with the living bacterium *P. fulva* SB1 (Figure 5 and Supplementary Table S10). As another similar perylenequinone toxin cercosporin produced by *Cercospora* could be activated by light to generate reactive oxygen species (ROS) for membrane damage and cell death of host plant cells (Daub and Ehrenshaft, 2000), we assumed that *P. fulva* SB1 could stimulate *Shiraia* for HA biosynthesis to produce more ROS for infection to bamboo tissues since HA was also reported as a fungal photosensitizer with ROS generation (Mulrooney et al., 2012). In contrast to *Pseudomonas* species, some isolates from the fruiting bodies such as *B. cereus* No. 1 were found to inhibit the accumulating of perylenequinone pigments completely (Figure 5 and Supplementary Table S10), suggesting a specific response of host fungus toward different bacteria rather than a general reaction. We investigated the influence of the living *B. cereus* No. 1 on fungal growth and HA biosynthesis (Figure 6). The growth inhibition and the suppression of HA production (intracellular biosynthesis and extracellular accumulation) were found in the co-cultures. Later, we also confirmed that the self-toxicity of the released HA could be alleviated by *B. cereus*, partly due to the biodegradation (Figures 11C,D). As far as the transcript changes of HA biosynthetic genes were concerned, the bacterial effects were also contrasting (Figure 7). *P. fulva* SB1 not only participated in the transcript induction on HA biosynthetic genes including *PKS*, *Omef*, *Mono*, *MCO*, and *ZFIF*, but also up-regulated *MFS* and *ABC* expression for HA exudation, resulting in the significant enhancement of HA production in the co-cultures. However, *B. cereus* served to suppress or silence the expression of those target genes. Taken together with the information on HA production, our results suggested that some *Shiraia*-associated bacteria could be important players in the regulation of fungal HA biosynthesis in the fruiting bodies.

Since HA has great potential in PDT for cancers and skin diseases (Mulrooney et al., 2012; Jin et al., 2013), extensive applications as strong antimicrobial agents (Shen et al., 2012), and food colorants in agricultural and feed industry (Su et al., 2011), mycelium cultures have been considered as an attractive alternative to the limited supply of wild *Shiraia* fruiting bodies in HA production. HA yield was achieved as 2.02 mg/g (dry solid substrate) in the solid cultures (Liang et al., 2009) and about 10–40 mg/L in liquid mycelium cultures (Liu et al., 2009). Thus, many strategies have been applied to enhance HA production in the mycelium cultures, including optimizing the cultural medium (Yang et al., 2013), elicitation (Cai et al., 2011; Du et al., 2013; Ma et al., 2019), and molecular engineering (Gao et al., 2018). In our previous study (Sun et al., 2017), the highest HA production (247.67 mg/L) was obtained under the elicitation of a repeated ultrasound. We also found that a light/dark shift (24:24 h) could enhance HA production by 73% over the dark control (Sun et al., 2018). In the present study, after an addition of *P. fulva* SB1 to the mycelium culture, the total HA production was increased by more than 3.25-fold (225.34 mg/L) of the control (Figure 10). Bacteria–fungi interaction has been utilized to induce or improve the metabolite production in mycelium cultures. Cueto et al. (2001) demonstrated that the induction of a new chlorinated benzophenone antibiotic, pestalone, was conducted in co-cultivation of a marine fungus *Pestalotia* sp. and a Gram-negative unicellular bacterium (strain CNJ-328). The addition of the same strain induced the production of new pimarane diterpenoids, libertellenones A–D in cultures of the marine-derived fungus *Libertella* sp. (Oh et al., 2005). An enhanced production (up to 78-fold) of known metabolites and three new natural products were identified only in co-cultures of the endophytic *Fusarium tricinctum* with the bacterium *B. subtilis* 168 trpC2 (Ola et al., 2013). Compared to other abiotic elicitors such as ultrasound, light–dark shift, Triton X-100, and red light (Lei et al., 2017; Sun et al., 2017, 2018; Ma et al., 2019), bacterial treatment is simple and easily prepared without the need for complex equipment. It is worth mentioning that bacterial co-culture at the desired condition exhibited no suppression of fungal biomass (Figure 10A), suggesting an effective strategy for improving HA production in mycelium cultures.

## Conclusion

In summary, this study presented the first assessment of the diversity of bacteria communities inhabiting in the fruiting body of *S. bambusicola* by using culture-dependent and -independent approaches. The data revealed that the predominant bacteria are *Bacillus* and *Pseudomonas*. It is an interesting finding that the associated bacteria from some *Pseudomonas* strains exhibited the capacity to stimulate HA biosynthesis in host *Shiraia* fungus, whereas some *Bacillus* strains suppressed fungal HA production significantly. These results revealed some physiological roles of the associated bacteria on the regulation of metabolites of host fungus in the fruiting bodies. To our best knowledge, this is the first report to show that live bacterium could regulate the biosynthesis of hypocrellins in *Shiraia*. Although the mechanism of the elicitation or inhibition on HA biosynthesis needs further investigation, the present study successfully provided a novel co-culture method for HA production. With the optimization of co-culture conditions or using the combined eliciting strategies, we believe that HA production in a large-scale culture of *Shiraia* could be improved greatly.

## Author Contributions

JW and YM were the recipients of funds, conceived the experiments, and prepared the manuscript. YM and LZ undertook experiments and data analysis. All authors have read and approved the final manuscript.

## Conflict of Interest Statement

The authors declare that the research was conducted in the absence of any commercial or financial relationships that could be construed as a potential conflict of interest.

## Footnotes

1 http://phylogeny.lirmm.fr/phylo_cgi/index.cgi

2 https://www.ncbi.nlm.nih.gov/genbank/

3 https://blast.ncbi.nlm.nih.gov/Blast.cgi

## References

[B1] Anthony M. B. Marc L. Bjoern U. (2014). Trimmomatic: a flexible trimmer for Illumina sequence data. *Bioinformatics* 30 2114–2120. 10.1093/bioinformatics/btu170 24695404 PMC4103590

[B2] Benucci G. M. N. Bonito G. M. (2016). The truffle microbiome: species and geography effects on bacteria associated with fruiting bodies of hypogeous Pezizales. *Microb. Ecol.* 72 4–8. 10.1007/s00248-016-0755-3 27026101

[B3] Cai Y. J. Liao X. R. Liang X. H. Ding Y. R. Sun J. Zhang D. B. (2011). Induction of hypocrellin production by Triton X-100 under submerged fermentation with *Shiraia* sp. *SUPER-H*168. *New Biotechnol.* 28 588–592. 10.1016/j.nbt.2011.02.001 21324385

[B4] Caporaso J. G. Lauber C. L. Walters W. A. Berg-Lyons D. Huntley J. Fierer N. (2012). Ultra-high-throughput microbial community analysis on the Illumina HiSeq and MiSeq platforms. *ISME J.* 6 1621–1624. 10.1038/ismej.2012.8 22402401 PMC3400413

[B5] Chen T. F. Jia X. M. Ma X. H. Lin H. P. Zhao Y. H. (2004). Phylogenetic study on *Shiraia bambusicola* by rDNA sequence analyses. *J. Basic Microbiol.* 44 339–350. 10.1002/jobm.200410434 15378525

[B6] Cho Y. S. Kim J. S. Crowley D. E. Cho B. G. (2003). Growth promotion of the edible fungus *Pleurotus ostreatus* by *Fluorescent pseudomonads*. *FEMS Microbiol. Lett.* 218 271–276. 12586403 10.1016/S0378-1097(02)01144-8

[B7] Citterio B. Malatesta M. Battistelli S. Marcheggiani F. Baffone W. Saltarelli R. (2001). Possible involvement of *Pseudomonas fluorescens* and *Bacillaceae* in structural modifications of *Tuber borchii* fruit bodies. *Can. J. Microbiol.* 47 264–268. 10.1139/cjm-47-3-264 11315117

[B8] Cueto M. Jensen P. R. Kauffman C. Fenical W. Lobkovsky E. Clardy J. (2001). Pestalone, a new antibiotic produced by a marine fungus in response to bacterial challenge. *J. Nat. Prod.* 64 1444–1446. 10.1021/np0102713 11720529

[B9] Dahm H. Wrotniak W. Strzelczyk E. Li C. Y. Bednarska E. (2005). Diversity of culturable bacteria associated with fruiting bodies of ectomycorrhizal fungi. *Phytopathol. Pol.* 38 51–62.

[B10] Danell E. Alström S. Ternström A. (1993). Pseudomonas fluorescens in association with fruit bodies of the ectomycorrhizal mushroom *Cantharellus cibarius*. *Mycol. Res.* 97 1148–1152. 10.1016/s0953-7562(09)80519-4

[B11] Daub M. E. Ehrenshaft M. (2000). The photoactivated *Cercospora* toxin cercosporin: contributions to plant disease and fundamental biology. *Annu. Rev. Phytopathol.* 38 461–490. 10.1146/annurev.phyto.38.1.461 11701851

[B12] DeSantis T. Z. Hugenholtz P. Larsen N. Rojas M. Brodie E. L. Keller K. (2006). Greengenes, a chimera-checked 16S rRNA gene database and workbench compatible with ARB. *Appl. Environ. Microbiol.* 72 5069–5072. 10.1128/aem.03006-05 16820507 PMC1489311

[B13] Di W. Zhen J. Lown J. W. (1990). Hypocrellins and their use in photosensitization. *Photochem. Photobiol.* 52 609–616. 10.1111/j.1751-1097.1990.tb01807.x 2284353

[B14] Du W. Liang Z. Q. Zou X. Han Y. F. Liang J. Yu J. P. (2013). Effects of microbial elicitor on production of hypocrellin by *Shiraia bambusicola*. *Folia Microbiol.* 58 283–289. 10.1007/s12223-012-0203-9 23229285

[B15] Gao R. J. Xu Z. C. Deng H. X. Guan Z. B. Liao X. R. Zhao Y. (2018). Enhanced hypocrellin production of *Shiraia* sp. SUPER-H168 by overexpression of alpha-amylase gene. *PLoS One* 13:e0196519. 10.1371/journal.pone.0196519 29723233 PMC5933700

[B16] Hu F. Li R. X. Li C. R. Fan M. Z. (2008). Hypocrellins produced by liquid fermentation of an anamorphic strain from *Shiraia bambusicola*. *J. Biol.* 25 44–47.

[B17] Jin S. Zhou L. J. Gu Z. J. Tian G. Yan L. Ren W. L. (2013). A new near infrared photosensitizing nanoplatform containing blue-emitting up-conversion nanoparticles and hypocrellin a for photodynamic therapy of cancer cells. *Nanoscale* 5 11910–11918. 10.1039/c3nr03515h 24129918

[B18] Kataoka R. Siddiqui Z. A. Kikuchi J. Ando M. Sriwati R. Nozaki A. (2012). Detecting nonculturable bacteria in the active mycorrhizal zone of the pine mushroom *Tricholoma matsutake*. *J. Microbiol.* 50 199–206. 10.1007/s12275-012-1371-7 22538647

[B19] Kreig N. R. Holt J. G. (1984). *Bergey’s Manual of Systematic Bacteriology*, Vol. I. Baltimore, MD: Williams and Wilkins.

[B20] Kumari D. Reddy M. S. Upadhyay R. C. (2013). Diversity of cultivable bacteria associated with fruiting bodies of wild Himalayan *Cantharellus* spp. *Ann. Microbiol.* 63 845–853. 10.1007/s13213-012-0535-3

[B21] Lei X. Y. Zhang M. Y. Ma Y. J. Wang J. W. (2017). Transcriptomic responses involved in enhanced production of hypocrellin A by addition of Triton X-100 in submerged cultures of *Shiraia bambusicola*. *J. Ind. Microbiol. Biotechnol.* 44 1415–1429. 10.1007/s10295-017-1965-5 28685359

[B22] Li Q. Chen C. Penttinen P. Xiong C. Zheng L. Huang W. (2016a). Microbial diversity associated with *Tricholoma matsutake* fruiting bodies. *Microbiol.* 85 531–539. 10.1134/s0026261716050106 26428733

[B23] Li Q. Li X. L. Chen C. Li S. H. Huang W. L. Xiong C. (2016b). Analysis of bacterial diversity and communities associated with *Tricholoma matsutake* fruiting bodies by barcoded pyrosequencing in Sichuan province, southwest China. *J. Microbiol. Biotechnol.* 26 89–98. 10.4014/jmb.1505.05008 26428733

[B24] Li W. Z. Godzik A. (2006). Cd-hit: a fast program for clustering and comparing large sets of protein or nucleotide sequences. *Bioinformatics* 22 1658–1659. 10.1093/bioinformatics/btl158 16731699

[B25] Liang X. H. Cai Y. J. Liao X. R. Wu K. Wang L. Zhang D. B. (2009). Isolation and identification of a new hypocrellin A-producing strain *Shiraia* sp. *SUPER-H*168. *Microbiol. Res.* 164 9–17. 10.1016/j.micres.2008.08.004 18809305

[B26] Liu Y. X. Liu Z. Y. Yang Y. L. Wongkaew S. (2009). Isolation, screening and confirmative identification of high hypocrellin A-producing *Shiraia bambusicola* isolates. *Khon. Kaen. Agric. J.* 37 357–364.

[B27] Lyon H. Holm I. Prentø P. Balslev E. (1995). Non-hazardous organic solvents in the paraffin-embedding technique: a rational approach. *Histochem. Cell Biol.* 103 263–269. 10.1007/bf01457410 7648401

[B28] Ma Y. J. Sun C. X. Wang J. W. (2019). Enhanced production of hypocrellin A in submerged cultures of *Shiraia bambusicola* by red light. *Photochem. Photobiol.* 95 812–822. 10.1111/php.13038 30338861

[B29] Mitchell T. K. Chilton W. S. Daub M. E. (2002). Biodegradation of the polyketide toxin cercosporin. *Appl. Environ. Microbiol.* 68 4173–4181. 10.1128/aem.68.9.4173-4181.2002 12200262 PMC124086

[B30] Mulrooney C. A. O’Brien E. M. Morgan B. J. Kozlowski M. C. (2012). Perylenequinones: isolation, synthesis, and biological activity. *Eur. J. Org. Chem.* 21 3887–3904. 10.1002/ejoc.201200184 24039544 PMC3770481

[B31] Nazir R. Warmink J. A. Boersma H. Elsas J. D. V. (2010). Mechanisms that promote bacterial fitness in fungal-affected soil microhabitats. *FEMS Microbiol. Ecol.* 71 169–185. 10.1111/j.1574-6941.2009.00807.x 20002182

[B32] O’Brien E. M. Morgan B. J. Mulrooney C. A. Carroll P. J. Kozlowski M. C. (2010). Perylenequinone natural products: total synthesis of hypocrellin A. *J. Org. Chem.* 75 57–68. 10.1021/jo901386d 19894741 PMC2798931

[B33] Oh D. C. Jensen P. R. Kauffman C. A. Fenical W. (2005). Libertellenones A-D: induction of cytotoxic diterpenoid biosynthesis by marine microbial competition. *Bioorg. Med. Chem.* 13 5267–5273. 10.1016/j.bmc.2005.05.068 15993608

[B34] Ola A. R. B. Thomy D. Lai D. W. Brötz-Oesterhelt H. Proksch P. (2013). Inducing secondary metabolite production by the endophytic fungus *Fusarium tricinctum* through coculture with *Bacillus subtilis*. *J. Nat. Prod.* 76 2094–2099. 10.1021/np400589h 24175613

[B35] Pent M. Põldmaa K. Bahram M. (2017). Bacterial communities in boreal forest mushrooms are shaped both by soil parameters and host identity. *Front. Microbiol.* 8:836. 10.3389/fmicb.2017.00836 28539921 PMC5423949

[B36] Poole E. J. Bending G. D. Whipps J. M. Read D. J. (2001). Bacteria associated with *Pinus sylvestris–Lactarius rufus* ectomycorrhizas and their effects on mycorrhiza formation *in vitro*. *New Phytol.* 151 743–751. 10.1046/j.0028-646x.2001.00219.x 33853249

[B37] Qi S. S. Fan Y. Gong Z. N. Yan S. Z. Zhao B. T. Chen S. L. (2015). Genetic diversity of *Shiraia bambusicola* from East China assessed using ISSR markers. *Biochem. Syst. Ecol.* 59 239–245. 10.1016/j.bse.2015.01.007

[B38] Reyon D. Tsai S. Q. Khayter C. Foden J. A. Sander J. D. Joung J. K. (2012). FLASH assembly of TALENs enables high-throughput genome editing. *Nat. Biotechnol.* 30 460–465. 10.1038/nbt.2170 22484455 PMC3558947

[B39] Sbrana C. Agnolucci M. Bedini S. Lepera A. Toffanin A. Giovannetti M. (2002). Diversity of culturable bacterial populations associated to *Tuber borchii* ectomycorrhizas and their activity on *T. borchii, mycelial growth*. *FEMS Microbiol. Lett.* 211 195–201. 10.1016/s0378-1097(02)00712-7 12076812

[B40] Sbrana C. Bagnoli G. Bedini S. Filippi C. Giovannetti M. Nuti M. P. (2000). Adhesion to hyphal matrix and antifungal activity of *Pseudomonas* strains isolated from *Tuber borchii* ascocarps. *Can. J. Microbiol.* 46 259–268. 10.1139/cjm-46-3-259 10749539

[B41] Schloss P. D. Westcott S. L. Ryabin T. Hall J. R. Hartmann M. Hollister E. B. (2009). Introducing mothur: open-source, platform-independent, community-supported software for describing and comparing microbial communities. *Appl. Environ. Microbiol.* 75 7537–7541. 10.1128/AEM.01541-09 19801464 PMC2786419

[B42] Shen X. Y. Cheng Y. L. Cai C. J. Fan L. Gao J. Hou C. L. (2014). Diversity and antimicrobial activity of culturable endophytic fungi isolated from moso bamboo seeds. *PloS One* 9:e95838. 10.1371/journal.pone.0095838 24759896 PMC3997407

[B43] Shen X. Y. Zheng D. Q. Gao J. Hou C. L. (2012). Isolation and evaluation of endophytic fungi with antimicrobial ability from *Phyllostachys edulis*. *Bangl. J. Pharmacol.* 7 249–257.

[B44] Singh M. P. Leighton M. M. Barbieri L. R. Roll D. M. Urbance S. E. Hoshan L. (2010). Fermentative production of self-toxic fungal secondary metabolites. *J. Ind. Microbiol. Biotechnol.* 37 335–340. 10.1007/s10295-009-0678-9 20033470

[B45] Splivallo R. Deveau A. Valdez N. Kirchhoff N. Frey-Klett P. Karlovsky P. (2015). Bacteria associated with truffle-fruiting bodies contribute to truffle aroma. *Environ. Microb.* 17 2647–2660. 10.1111/1462-2920.12521 24903279

[B46] Su Y. J. Si S. H. Qiao L. W. Cai Y. J. Xu Z. M. Yang Y. J. (2011). The effect of a hypocrellin A enriched diet on egg yolk quality and hypocrellin A distributions in the meat of laying hens. *Eur. Food Res. Technol.* 232 935–940. 10.1007/s00217-011-1461-5

[B47] Su Y. J. Yin X. Y. Rao S. Q. Cai Y. J. Reuhs B. Yang Y. J. (2009). Natural colourant from *Shiraia bambusicola*: stability and antimicrobial activity of hypocrellin extract. *Int. J. Food Sci. Technol.* 44 2531–2537. 10.1111/j.1365-2621.2009.02080.x

[B48] Sun C. X. Ma Y. J. Wang J. W. (2017). Enhanced production of hypocrellin A by ultrasound stimulation in submerged cultures of *Shiraia bambusicola*. *Ultrason. Sonochem.* 38 214–224. 10.1016/j.ultsonch.2017.03.020 28633821

[B49] Sun C. X. Ma Y. J. Wang J. W. (2018). Improved hypocrellin A production in *Shiraia bambusicola* by light–dark shift. *J. Photochem. Photobiol. B Biol.* 182 100–107. 10.1016/j.jphotobiol.2018.04.004 29656218

[B50] Varese G. C. Portinaro S. Trotta A. Scannerini S. Luppi A. M. Martinotti G. (1996). Bacteria associated with *Suillus grevillei* sporocarps and ectomycorrhizae and their effects on *in vitro* growth of the mycobiont. *Symbiosis* 21 129–147.

[B51] Wan X. Y. Chen Y. T. (1981). Hypocrellin A, a new drug for photochemotherapy. *Chin. Sci. Bull.* 26 1040–1041.

[B52] Wang Q. Garrity G. M. Tiedje J. M. Cole J. R. (2007). Naive Bayesian classifier for rapid assignment of rRNA sequences into the new bacterial taxonomy. *Appl. Environ. Microbiol.* 73 5261–5267. 10.1128/aem.00062-07 17586664 PMC1950982

[B53] Wang X. M. Yang B. Wang H. W. Yang T. Ren C. G. Zheng H. L. (2015). Consequences of antagonistic interactions between endophytic fungus and bacterium on plant growth and defense responses in *Atractylodes lancea*. *J. Basic Microbiol.* 55 659–670. 10.1002/jobm.201300601 24293321

[B54] Wu L. Y. Wen C. Q. Qin Y. J. Yin H. Q. Tu Q. C. Nostrand J. D. V. (2015). Phasing amplicon sequencing on Illumina Miseq for robust environmental microbial community analysis. *BMC Microbiol.* 15:125. 10.1186/s12866-015-0450-4 26084274 PMC4472414

[B55] Xiang Q. J. Luo L. H. Liang Y. H. Chen Q. Zhang X. P. Gu Y. F. (2017). The diversity, growth promoting abilities and anti-microbial activities of bacteria isolated from the fruiting body of *Agaricus bisporus*. *Pol. J. Microbiol.* 66 201–207. 10.5604/01.3001.0010.7837 28735315

[B56] Yang H. L. Xiao C. Ma W. He G. (2009). The production of hypocrellin colorants by submerged cultivation of the medicinal fungus *Shiraia bambusicola*. *Dyes Pigm.* 82 142–146. 10.1016/j.dyepig.2008.12.012

[B57] Yang Y. C. Ding Y. R. Liao X. R. Cai Y. J. (2013). Purification and characterization of a new laccase from *Shiraia* sp. *SUPER-H*168. *Process Biochem.* 48 351–357. 10.1016/j.procbio.2012.12.011

[B58] Yuan Z. S. Liu F. Zhang G. F. (2015). Isolation of culturable endophytic bacteria from Moso bamboo (*Phyllostachys edulis*) and 16S rDNA diversity analysis. *Arch. Biol. Sci.* 67 1001–1008. 19224269

[B59] Yurkov A. Krüger D. Begerow D. Arnold N. Tarkka M. T. (2012). Basidiomycetous yeasts from boletales fruiting bodies and their interactions with the mycoparasite *Sepedonium chrysospermum* and the host fungus *Paxillus*. *Microb. Ecol.* 63 295–303. 10.1007/s00248-011-9923-7 21833540

[B60] Zagryadskaya Y. A. Lysak L. V. Sidorova I. I. Aleksandrova A. V. Voronina E. Y. (2013). Bacterial complexes of the fruiting bodies and hyphosphere of certain basidiomycetes. *Biol. Bull.* 40 358–364. 10.1134/s106235901304016x 24459845

[B61] Zhao N. Lin X. Qi S. S. Luo Z. M. Chen S. L. Yan S. Z. (2016). *De novo* transcriptome assembly in *Shiraia bambusicola* to investigate putative genes involved in the biosynthesis of hypocrellin A. *Int. J. Mol. Sci.* 17:311. 10.3390/ijms17030311 26927096 PMC4813174

[B62] Zhen J. Di W. (1995). Novel therapeutic and diagnostic applications of hypocrellins and hypericins. *Photochem. Photobiol.* 61 529–539. 10.1111/j.1751-1097.1995.tb09903.x 7568399

