# Supplementary material for: Bacteria Associated With Shiraia Fruiting Bodies Influence Fungal Production of Hypocrellin A
Source: Front Microbiol. 2019 Sep 11;10:2023. doi: 10.3389/fmicb.2019.02023 (PMC6749022; doi:10.3389/fmicb.2019.02023)
Supplement: Supplementary file 1 [file Data_Sheet_1.PDF]

Frontiers in Microbiology
Supplementary Information

Bacteria associated with *Shiraia* fruiting bodies influence fungal production of hypocrellin A

Yan Jun Ma [1] ·Li Ping Zheng [2] ·Jian Wen Wang [1] *

✉ Jian Wen Wang
jwwang@suda.edu.cn, bcjwwang@gmail.com

[1] College of Pharmaceutical Sciences, Soochow University, Suzhou 215123, China
[2] Department of Horticultural Sciences, Soochow University, Suzhou 215123, China

TABLE S1| The isolated HA-yielding *Shiraia* strains from the fruiting body.
TABLE S2| The primers of some HA biosynthetic genes and the internal reference gene used for qRT-PCR. F: forward primer, R: reverse primer.
TABLE S3| Taxonomic classification of 31 bacterial isolates from *Shiraia* fruiting bodies (See in Excel file).
TABLE S4| 16S rDNA gene sequence analysis of companion bacteria isolated from *Shiraia* fruiting body based on BLAST analysis in the NCBI database.
TABLE S5| Physiological and biochemical characteristics of representative companion bacteria isolated from *Shiraia* fruiting bodies.
TABLE S6| Sequence and diversity statistics of bacterial 16S rDNA genes obtained from this study.
TABLE S7| Taxonomic classification of OTUs detected from the fruiting bodies with total read numbers (See in Excel file).
TABLE S8| The OTU taxonomy of bacterial community composition of the fruiting body.
TABLE S9| Taxonomic classification of OTUs at the genus level obtained from the fruiting bodies (See in Excel file).
TABLE S10| Effect of live bacterium on HA production of their host fungus *Shiraia* sp. S9 in solid-state cultures.

FIGURE S1| The morphology and structure observation of the fruiting body.
FIGURE S2| The agarose gel electrophoresis of ITS rDNA PCR products (A) and phylogenetic tree of sequencing results of strain S9 (B).
FIGURE S3| The colony morphology of 31 bacteria strains isolated from the fruiting body.
FIGURE S4| The effects of the cultivable bacteria on the growth and red pigment secretion of *Shiraia* sp. S9.
FIGURE S5| (A) Macroscopic colony appearance of SB1 strain on LB agar plate for 24 h. (B) Phylogenic tree of *P. fulva* SB1 and its phylogenetically related reference strains based on 16S rDNA sequences.

TABLE S1| The isolated HA-yielding *Shiraia* strains from the fruiting body.

Strain No.	HA content (mg/cm^2)	Nearest match in NCBI database and accession No.	Identity (%)
S1	0.26 ± 0.03	*Shiraia* sp. (JN198483.1)	99
S3	0.49 ± 0.12	*Shiraia* sp. (EU267793.1)	99
S9	2.96 ± 0.14	*Shiraia* sp. (EU267793.1)	94
S14	0.18 ± 0.01	*Shiraia* sp. (AY425966.2)	99
S23	1.16 ± 0.23	*Shiraia* sp. ML-2004 (AY425966.2)	99
S25	1.08 ± 0.15	*Shiraia* sp. isolate L34 (MH237669.1)	99

TABLE S2| The primers of some HA biosynthetic genes and the internal reference gene used for qRT-PCR. F: forward primer, R: reverse primer.

Gene name	Gene description	Sequence
18S	Reference gene	F: 5'-ACGCAGCGAAATGCGATAAG-3'<br>R: 5'-CAAATTGTGCTGCGCTCCAA-3'
MFS	*Shiraia* sp. slf14 major facilitator superfamily transporter [AIW00660.1]	F: 5'-TCCATTGTTCCAGGCGTACC-3'<br>R: 5'-TATGCCTCGCCGTTCTTTGT-3'
PKS	*Shiraia* sp. slf14 polyketide synthase [AIW00658.1]	F: 5'-GCTGTCCTGAAACGACTGGA-3'<br>R: 5'-CGAATATCGGGCACGTCTGA-3'
Mono	*Shiraia* sp. slf14 monooxygenase (hydroxylase) [AIW00664.1]	F: 5'-GGACGATTCCACGCGATTTG-3'<br>R: 5'-CATGGCTGACCGCATCATTG-3'
ABC	ATP-binding cassette transporter 1 [P41233.4]	F: 5'-CATGTCTCCCGACCTCATCG-3'<br>R: 5'-AAGCGCAGTGTTTCGTTGAC-3'
FAD	*Shiraia* sp. slf14 FAD/FMA-dependent oxidoreductase [AIW00665.1]	F: 5'-GATGGTTGCGTTGGCAAGTT-3'<br>R: 5'-GCTTCCCACCCATACGACAA-3'
MCO	Multicopper oxidase [XP_007708085.1]	F: 5'-CCCATCAAACCATTCGTCGC-3'<br>R: 5'-ATTTTGTGGCCGAGGTCCAT-3'
ZFIF	Zinc finger transcription factor 37 [Q5A4F3.1]	F: 5'-TACGATGAGCGCCAACAACT-3'<br>R: 5'-CTGCATGGCTATCGGGGATT-3'
Omef	*Shiraia* sp. slf14 *O*-methyltransferase [AIW00661.1]	F: 5'-GCTGGTGGACCTCTCCTTTC-3'<br>R: 5'-AGCGTGCCTTCAGGTAGTTC-3'

TABLE S3| Taxonomic classification of 31 bacterial isolates from *Shiraia* fruiting bodies (See in Excel file).

TABLE S4| 16S rDNA gene sequence analysis of companion bacteria isolated from *Shiraia* fruiting body based on BLAST analysis in the NCBI database.

Strain No.	Nearest match in NCBI database and accession No.	Query coverage (%)	Identity (%)
1	*Bacillus cereus* (EU621383.1)	97	97
2	*Brevibacterium* sp. (KU588052.1)	98	97
3	*Rhodococcus kroppenstedtii* (JN873342.1)	99	96
4	*Bacillus cereus* (HM752769.1)	98	98
5	*Bacillus* sp. (KY476269.1)	93	98
6	*Staphylococcus capitis* (JX094948.1)	93	97
7	*Bacillus tequilensis* (JF411311.1)	95	97
8	*Pseudomonas putida* (KX101237.1)	99	97
9	*Brevibacterium* sp. (KU588052.1)	97	97
10	*Bacillus* sp. (KM114620.1)	98	97
11	*Pseudomonas fulva* (AB681094.1)	98	99
12	*Pseudomonas fulva* (FJ972539.1)	94	97
13	*Aeromonas hydrophila* (KY496300.1)	99	96
14	*Staphylococcus equorum* (KP400528.1)	99	96
15	*Bacillus cereus* (EU621383.1)	96	97
16	*Bacillus subtilis* (KU904292.1)	95	97
17	*Bacillus cereus* (KX768300.1)	95	96
18	*Bacillus safensis* (KX269837.1)	93	98
19	*Bacillus anthracis* (JX307685.1)	98	97
20	*Pseudomonas parafulva* (KX345930.1)	99	96
21	*Escherichia coli* (KU204888.1)	99	96
22	*Bacillus* sp. (LC099946.1)	96	97
23	*Pseudomonas putida* (KX101237.1)	99	97
24	*Pseudomonas putida* (KM079616.1)	94	96
25	*Cupriavidus respiraculi* (AY860241.1)	94	98
26	*Bacillus cereus* (HM752769.1)	98	98
27	*Bacillus* sp. (FJ863099.1)	95	98
28	*Microbacterium oxydans* (EU714339.1)	98	97
29	*Enterobacter asburiae* (HQ455820.1)	99	97
30	*Staphylococcus aureus* (KJ643929.1)	98	96
31	*Bacillus subtilis* (KF641789.1)	98	97

TABLE S5| Physiological and biochemical characteristics of representative companion bacteria isolated from *Shiraia* fruiting bodies.

Classification (number)	Gram stain	Glucose utilization	Starch hydrolysis	Citrate untilization	Nitrate reduction	Oxidase test	Catalase test	GL test
Bacillus cereus (5)	+	+	+	-	+	-	+	+
Brevibacterium sp. (2)	+	+	-	+	+	-	+	+
Rhodococcus kroppenstedtii (1)	+	+	-	-	+	-	+	+
Staphylococcus capitis (1)	+	+	+	-	+	-	+	+
Bacillus tequilensis (1)	+	+	+	+	-	+	+	+
Bacillus sp. (4)	+	+	-	+	+	-	+	+
Pseudomonas putida (3)	-	+	-	+	+	+	+	-
Pseudomonas fulva (2)	-	+	-	+	-	+	+	+
Aeromonas hydrophila (1)	-	+	+	+	+	+	+	+
Staphylococcus equorum (1)	+	+	+	-	+	-	+	+
Bacillus subtilis (2)	+	+	+	+	+	-	+	+
Bacillus safensis (1)	+	+	-	+	+	+	+	+
Bacillus anthracis (1)	+	+	-	+	+	-	+	+
Pseudomonas parafulva (1)	-	+	-	+	-	+	+	+
Escherichia coli (1)	-	+	-	-	+	-	+	-
Cupriavidus respiraculi (1)	-	+	-	-	+	-	+	-
Microbacterium oxydans (1)	+	-	-	-	+	-	+	-
Enterobacter asburiae (1)	-	+	-	+	-	-	-	+
Staphylococcus aureus (1)	+	+	+	-	+	+	+	+

(+) positive reaction; (-) negative reaction; (GL) gelatin liquefaction; (MR) methyl red.

TABLE S6| Sequence and diversity statistics of bacterial 16S rDNA genes obtained from this study.

Sample name	Clean Reads [a]	OTU [b]	Shannon Wiener index [c]	Chao index [d]
The fruiting body	37610	723	4.468	806.817

a: The number of reads that passed quality control. b: Operational taxonomic unit.

c: Microbial diversity. d: Species abundance index.

TABLE S7| Taxonomic classification of OTUs detected from the fruiting bodies with total read numbers (See in Excel file).

TABLE S8| The OTU taxonomy of bacterial community composition of the fruiting body.

Sample name	Phylum	Class	Order	Family	Genus
S. Bambusicola fruiting body	30	84	149	244	364

TABLE S9| Taxonomic classification of OTUs at the genus level obtained from the fruiting bodies (See in Excel file).

TABLE S10| Effect of live bacterium on HA production of their host fungus *Shiraia* sp. S9 in solid-state cultures. A small piece (5 mm × 5 mm) of S9 strain was placed in the center of 10-cm PDA plate at 28°C for 4 d. The single colony of bacterium was inoculated in LB at 37°C on a rotary shaker at 200 rpm for 12 h. Then bacterial suspension (10 μL) was streaked in two parallel straight lines on PDA at 28°C for 10 d, approximately 7 cm apart from each other. Values are mean ± SD from three independent experiments. *$p < 0.05$, **$p < 0.01$ versus control group. ND means not detected.

Strain No.	Nearest match in NCBI database and accession No.	HA content (mg/cm^2)
Control	*Shiraia* sp. S9	2.96 ± 0.14
1	*Bacillus cereus* (EU621383.1)	ND
2	*Brevibacterium* sp. (KU588052.1)	0.62 ± 0.06**
3	*Rhodococcus kroppenstedtii* (JN873342.1)	1.19 ± 0.12**
4	*Bacillus cereus* (HM752769.1)	0.36 ± 0.02**
5	*Bacillus* sp. (KY476269.1)	0.83 ± 0.02**
6	*Staphylococcus capitis* (JX094948.1)	2.64 ± 0.31
7	*Bacillus tequilensis* (JF411311.1)	1.01 ± 0.13**
8	*Pseudomonas putida* (KX101237.1)	3.92 ±0.08*
9	*Brevibacterium* sp. (KU588052.1)	0.81 ± 0.27*
10	*Bacillus* sp. (KM114620.1)	1.12 ±0.01**
11	*Pseudomonas fulva* (AB681094.1)	6.18 ± 0.05**
12	*Pseudomonas fulva* (FJ972539.1)	4.07 ± 0.16**
13	*Aeromonas hydrophila* (KY496300.1)	3.12 ± 0.04
14	*Staphylococcus equorum* (KP400528.1)	2.80 ± 0.19
15	*Bacillus cereus* (EU621383.1)	ND
16	*Bacillus subtilis* (KU904292.1)	0.57 ± 0.03**
17	*Bacillus cereus* (KX768300.1)	0.28 ± 0.14**
18	*Bacillus safensis* (KX269837.1)	ND
19	*Bacillus anthracis* (JX307685.1)	ND
20	*Pseudomonas parafulva* (KX345930.1)	4.15 ± 0.18**
21	*Escherichia coli* (KU204888.1)	3.26 ± 0.25
22	*Bacillus* sp. (LC099946.1)	ND
23	*Pseudomonas putida* (KX101237.1)	3.77 ± 0.02*
24	*Pseudomonas putida* (KM079616.1)	4.01 ± 0.09**
25	*Cupriavidus respiraculi* (AY860241.1)	2.88 ± 0.12
26	*Bacillus cereus* (HM752769.1)	0.29 ± 0.03**
27	*Bacillus* sp. (FJ863099.1)	0.72 ± 0.15**
28	*Microbacterium oxydans* (EU714339.1)	3.18 ± 0.26
29	*Enterobacter asburiae* (HQ455820.1)	1.81 ± 0.14*
30	*Staphylococcus aureus* (KJ643929.1)	3.00 ± 0.18
31	*Bacillus subtilis* (KF641789.1)	0.12 ± 0.00**

FIGURE S1

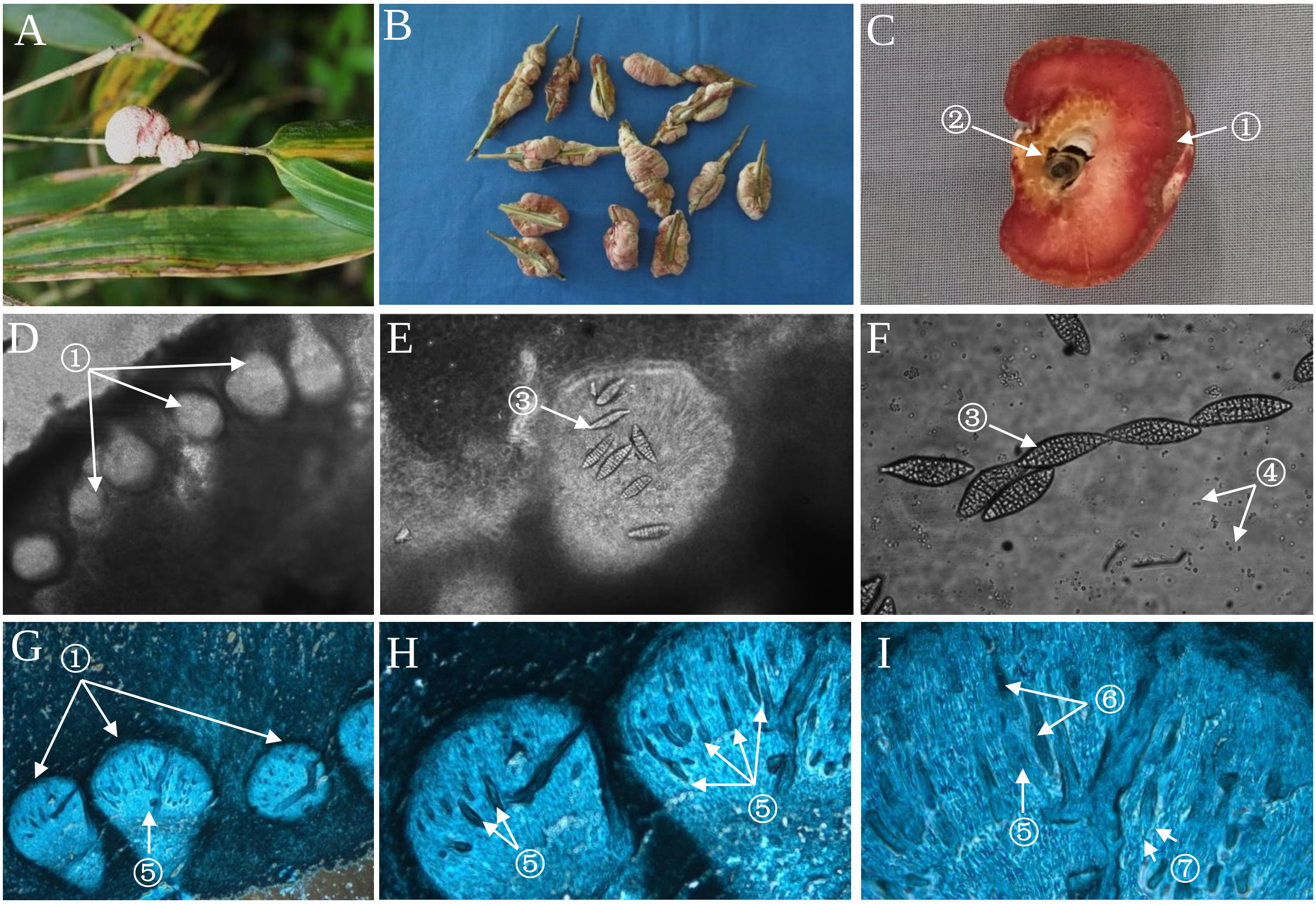

FIGURE S1| The morphology and structure observation of the fruiting body. The *Shiraia* stroma on the host bamboo branches (A and B). The stromal section (C). The morphology of fruiting body under light microscope (D, 100 ×; E, 200 ×; F, 400 ×). The observation of fruiting body under light microscope (G, 100 ×; H, 200 ×; I, 400 ×) ① Perithecium; ② Fiber of bamboo; ③ Ascospore; ④ Conidium; ⑤ Ascus; ⑥ Immature ascospore; ⑦ Paraphyses.

FIGURE S2

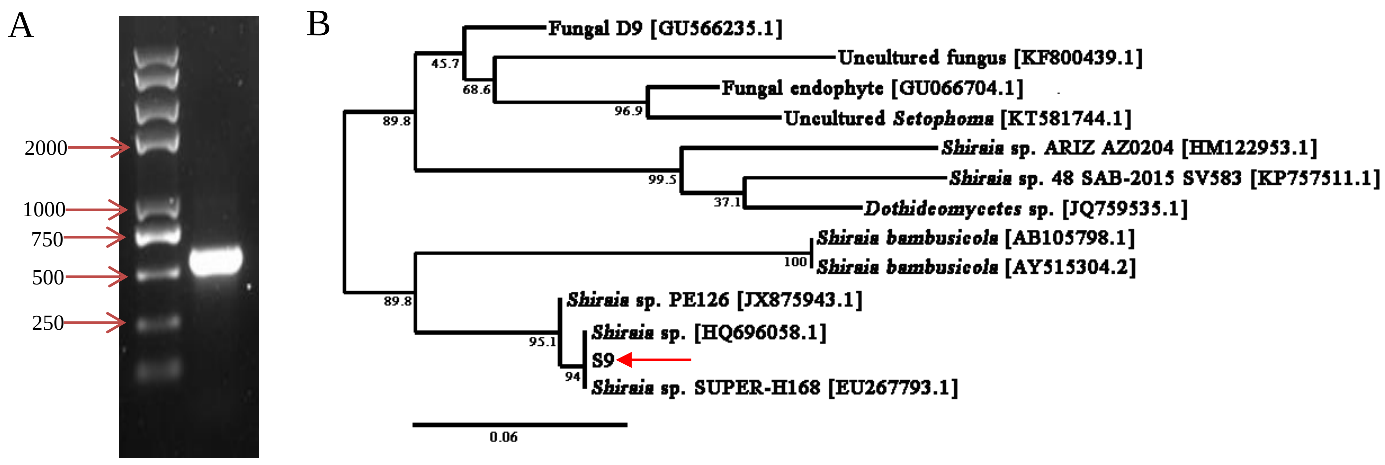

FIGURE S2| The agarose gel electrophoresis of ITS rDNA PCR products (A) and phylogenetic tree of sequencing results of strain S9 (B).

FIGURE S3

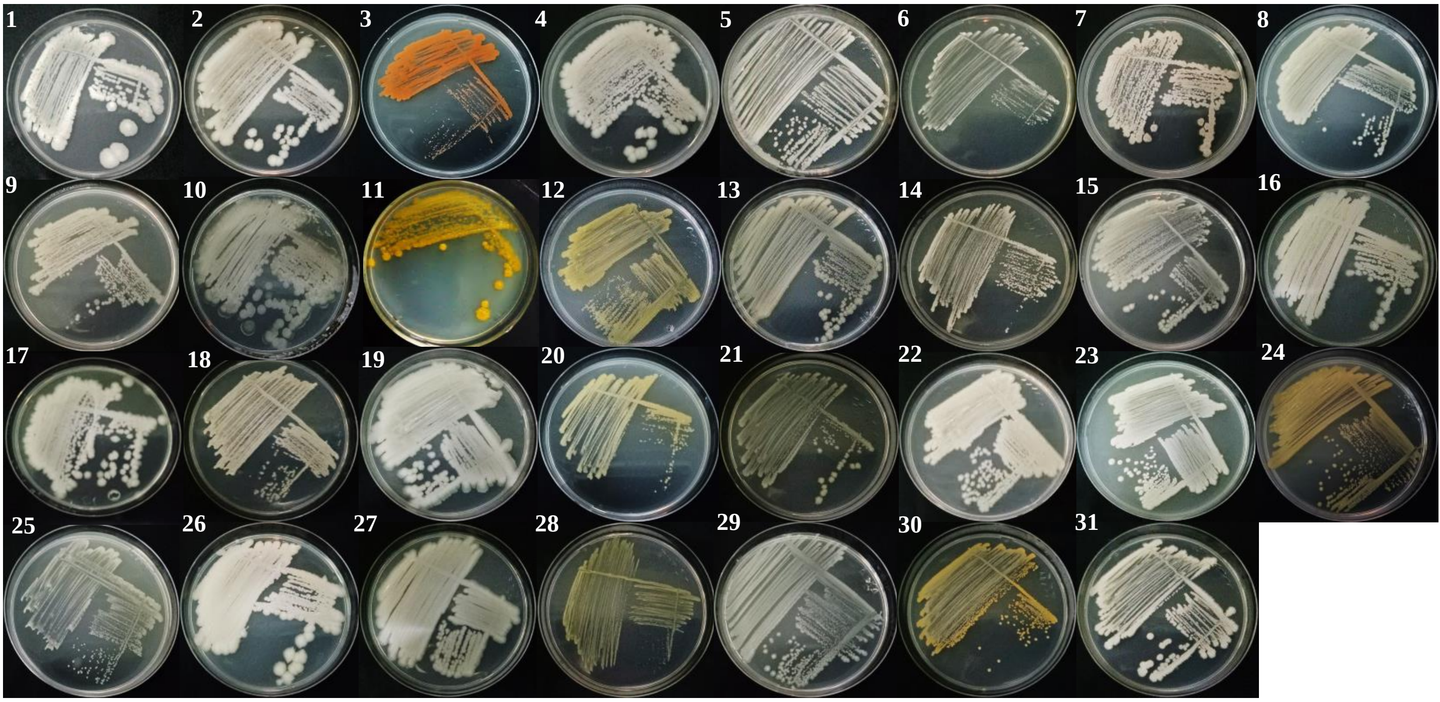

FIGURE S3| The colony morphology of 31 bacteria strains isolated from the fruiting body.

FIGURE S4

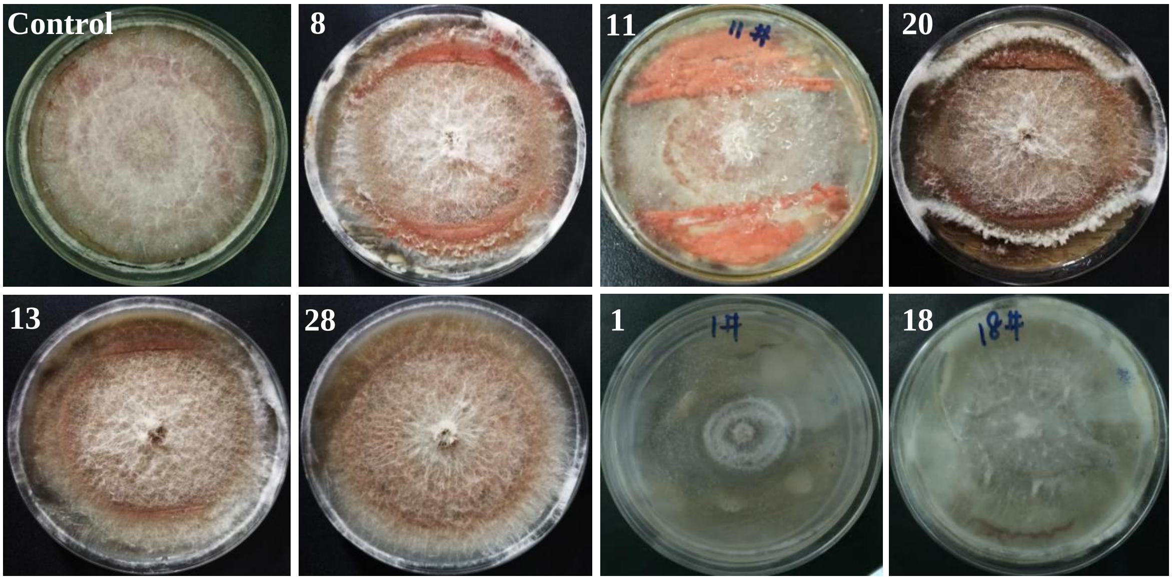

FIGURE S4| The effects of the cultivable bacteria on the growth and red pigment secretion of *Shiraia* sp. S9. A small piece (5 mm × 5 mm) of the strain was placed in the center of 10-cm PDA plate at 28°C for 4 d. The single colony of bacterium was inoculated in LB at 37°C on a rotary shaker at 200 rpm for 12 h. Then bacterial suspension (10 µL) was streaked in two parallel straight lines on PDA, approximately 7 cm apart from each other. After incubation for 10 days, the S9 colony morphology was photographed.

FIGURE S5

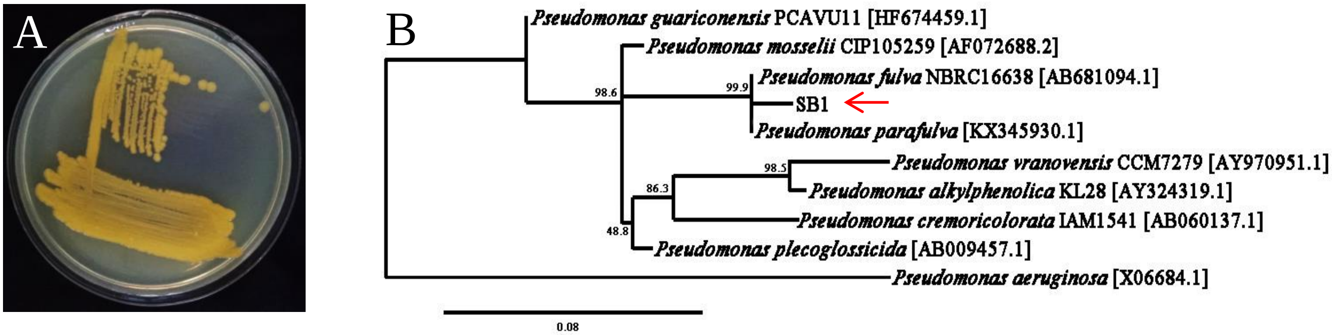

FIGURE S5| (A) Macroscopic colony appearance of SB1 strain on LB agar plate for 24 h. (B) Phylogenic tree of *P. fulva* SB1 and its phylogenetically related reference strains based on 16S rDNA sequences. The branch length is proportional to the number of substitutions per site. Bar, 0.08 substitutions per nucleotide position.
